# Cardiolipin-Specific Targeting of Melittin via Arg22 in Lipid Membranes: A Natural Toxin-Based Strategy for Treating Mitochondrial Dysfunction in Aging and Disease

**DOI:** 10.3390/toxins18100432

**Published:** 2026-10-08

**Authors:** Safy M. Sh. M. Abaza, Jiaqi Zhou, Ziqin Wang, Zanyu Pan, Haoran Ma, Yingdi Sun, Ruben K. Dagda, Edward S. Gasanoff

**Affiliations:** 1 Advanced STEM Research Center, Beijing Chaoyang Kaiwen Academy, Beijing 100018, China; safyabaza200504@gmail.com (S.M.S.M.A.); 2008290081@cy.kaiwenacademy.cn (J.Z.); 1806010027@cy.kaiwenacademy.cn (Z.W.); 2108280047@cy.kaiwenacademy.cn (Z.P.); 1806010005@cy.kaiwenacademy.cn (H.M.); 1703000008@cy.kaiwenacademy.cn (Y.S.); 2 Department of Pharmacology, University of Nevada Medical School, Reno, NV 89557, USA; rdagda@med.unr.edu; 3 Belozersky Institute of Physico-Chemical Biology, M.V. Lomonosov Moscow State University, Moscow 119991, Russia

**Keywords:** melittin, cardiolipin, membrane-mediated proton adsorption, non-bilayer lipid structures, mitochondrial ATP-synthase

## Abstract

Bee venom melittin is a small protein with significant pharmacological potential. In this study, AutoDock simulations identified Arg22 as a key residue of melittin responsible for targeting the phosphate groups of cardiolipin (CL). Recombinant melittin with the native amino acid sequence, as well as a modified version with Arg22 substituted by Gly22, were cloned, expressed, and purified. The effects of both melittins on proton adsorption in CL-containing membranes, mitochondrial ATP production, and the formation of non-bilayer lipid structures were examined. Based on the array of computational and experimental data obtained in this work, together with published findings, we propose that Arg22, in tandem with Lys23, targets and neutralizes CL bound to conserved Lys7 and Lys43 of the ATP synthase rotor, thereby detaching CL from the rotor. These events promote a conical shape of CL, which triggers the formation of inverted micelles that entrap protons from the F_o_ subunit and transfer them to the proton pump of a respiratory complex. This study advances both the conceptual framework of mitochondrial energetics and the translational application of natural toxins. Specifically, the findings support the development of new pharmaceuticals aimed at counteracting the loss of mitochondrial energy production that occurs with aging and pathological conditions.

## 1. Introduction

Melittin is a small membrane-active polypeptide from bee venom made of six basic, five polar and 15 hydrophobic amino acid residues [1,2]. By targeting biological membranes melittin induces a broad range of pharmacological effects including antimicrobial [3], antiarthritic [4], anti-inflammatory [5], and anticancer [6,7,8,9,10,11] reactions. Direct interaction of melittin with membranes of cells and organelles triggers changes in membrane structure resulting in membrane pores and membrane fusion [12,13] which may consequently induce aggregation of membrane proteins [14], and induction of hormone secretion [15] and changes in membrane potential [16].

By affecting the structure of cell membranes, melittin—similarly to cobra venom cytotoxins—modulates the activities of membrane-associated enzymes, including phospholipase A_2_ [17], phospholipase D [18], phospholipase C [19], protein kinase C [20], G-proteins [21], and adenylate cyclase [22]. This modulation results in a broad range of consequences, manifesting either as normal physiological processes in healthy cells or as the development of pathological processes that can lead to disease [23].

Non-enzymatic membrane-active small cationic proteins from insect and snake venom, such as bee venom melittin and cobra venom cytotoxins, along with phospholipase A_2_ enzymes, have been widely used to probe the structure and function of model lipid and biological membranes [2,23,24,25,26,27,28,29]. Synergistic effects in disrupting the bilayer organization of model lipid membranes and cell plasma membranes have been investigated in studies of the joint action of snake venom phospholipases A_2_ with either bee melittin [17,23,24] or cobra cytotoxins [25]. These efforts aim not only to elucidate the molecular mechanisms underlying the antimicrobial and anticancer effects of venom cationic membrane-active toxins, but also to understand the molecular events involved in the development of arthritis and inflammation [25].

Recent research over the past decade has revealed that cationic membrane-active proteins from cobra and bee venom, including cobra cytotoxins and melittin, target cardiolipin (CL) in model and biological membranes and can induce non-bilayer structures in CL-containing membranes [23,26,30,31], which in turn facilitate mitochondrial ATP synthase activity at low cationic protein concentrations [31,32]. Notably, at very low concentrations of melittin that produce no appreciable effect on inner mitochondrial membrane (IMM) structure or the rate of ATP synthase, the addition of short-chain alcohols synergistically enhances both the formation of non-bilayer lipid structures around melittin and the increase in ATP synthase activity [23].

A very recent study revealed that a protein fraction from bee venom can enhance proton absorption by membranes composed of myelin sheath phospholipids [33], in a manner similar to that observed in model myelin membranes enriched with proteins from *Latrodectus pallidus* spider venom, which share high sequence homology with myelin basic proteins [34]. Notably, spider venom proteins not only increase the proton adsorbing capacity of myelin membranes but also induce the formation of non-bilayer phases in these membranes [34] which normally leads to increase in mitochondrial ATP synthase activity as has been shown in parallel experiments with melittin and cobra cytotoxins [23,26,30,31,32].

These findings hold significant pharmacological relevance, as they align with novel concepts of bioenergetic mechanisms governing cellular energy accumulation and storage via adsorption of protons by protein–lipid complexes on a mitochondrial and extra-mitochondrial membrane surfaces as described in details in recent review articles [35,36]. The authors of these review articles along with other researchers suggest that protein–lipid complexes acting as proton energy capacitors could mitigate bioenergetic deficits associated with aging and/or disease [33,35,36].

The aim of this work was to identify the key amino acid residue of melittin that drives its attraction to CL—the principal phospholipid of mitochondria, which plays a crucial role in supporting most, if not all, functional activities of mitochondria mediated by cristae [30,37,38,39]. We employed AutoDock Vina version 4.2 for computer simulations of melittin interaction with CL to determine the best tentative binding sites for CL on the molecular surface of melittin. The results featured the Arg22 residue of melittin in virtually all tentative binding sites, with Arg22 forming polar-ionic and hydrogen intermolecular bonds with the two phosphate groups and polar moieties of CL. We then used PyMOL^TM^ 2.5.5 to modify the amino acid sequence of melittin by replacing Arg22 with glycine—an amino acid residue lacking an R-group side chain. AutoDock simulations of the modified melittin with CL revealed a pattern of CL binding that was entirely different from that observed for native melittin.

We subsequently produced two recombinant melittins (r-melittins)—one with the native sequence and another with the modified sequence (Arg22 → Gly22)—using a recombinant protein expression protocol. Further experiments with both recombinant native and modified melittins showed that they differ in their ability to modulate mitochondrial ATP synthase activity, as well as in their capacity to affect proton adsorption and trigger non-bilayer transitions in CL-containing membranes.

Overall, the results obtained in this study demonstrate the effectiveness of combining computational and experimental approaches to drive research efforts toward the development of novel pharmaceuticals based on natural proteins, with potential applications in treating cellular energy decline caused by aging and/or disease.

## 2. Results

### 2.1. AutoDock Simulation of Native and Modified Melittin Interaction with Cardiolipin

**Figure 1:**
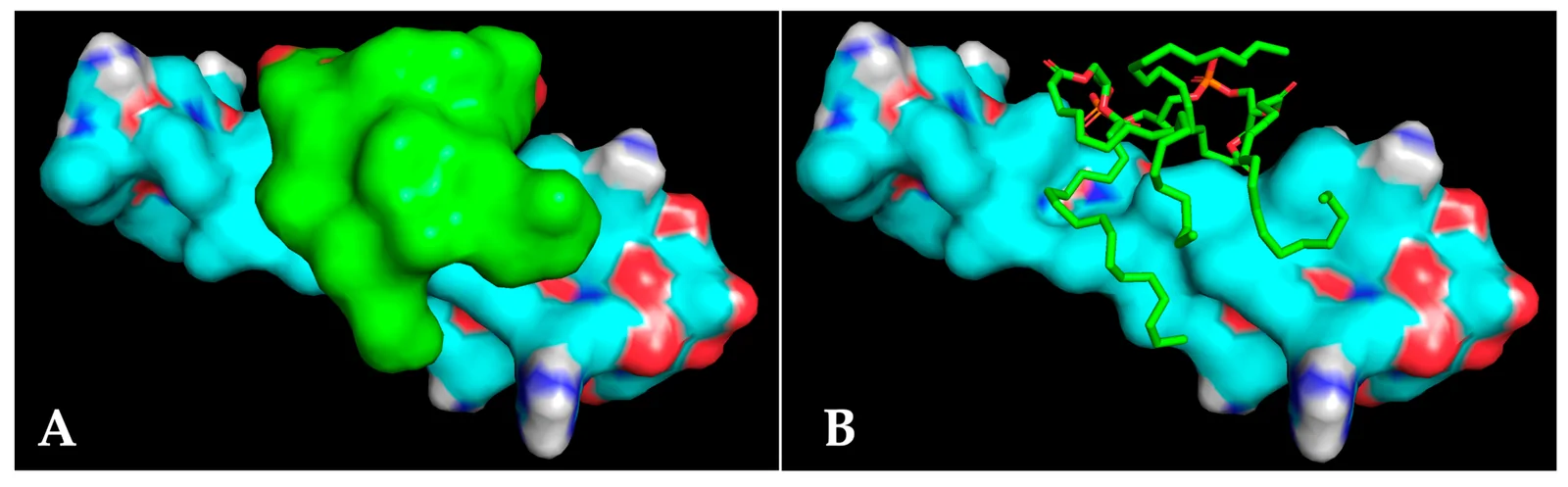
Binding site #1 for CL on the molecular surface of native melittin, as predicted by AutoDock Vina simulation. Melittin and CL are shown in molecular surface representation in (**A**), while in (**B**), melittin is shown in molecular surface representation and CL is rendered in stick representation. In the molecular surface representation, carbon of melittin is shown in emerald and carbon of CL is shown in green, oxygen in red, hydrogen in white, and nitrogen in blue. In the stick representation, carbon is shown in green, phosphorus in orange, and oxygen in red. Hydrogen atoms in the stick representation are omitted for visual clarity.

We used CL as a ligand and melittin as a receptor in our docking simulations, following the protocol described previously [40], to identify the amino acid residue of melittin most frequently involved in intermolecular bonds with the charged and polar moieties of CL. Table 1 presents the nine best binding sites with the highest affinities for CL binding to the molecular surface of melittin. The negative values of binding affinities indicate an exothermic process of intermolecular bond formation. As shown in Table 1, AutoDock identified three sites on the molecular surface of melittin (binding sites #1, #4, and #8) where the alkyl chains of CL bind to hydrophobic amino acid residues of melittin, forming extensive areas of non-polar London dispersion forces of attraction (Figure 1).

**Table 1:** Binding site affinity energies, polar and charged moieties of native melittin and the CL polar head, and types of intermolecular bonds predicted by AutoDock Vina simulation. Notations for the polar and charged groups in the CL polar head are provided in Figure 2. For binding site #3, the polar and charged moieties of melittin are labeled with numbers, and those of CL are labeled with letters, following the same labeling scheme used in Figure 3.

Number of Binding Site and Binding Affinity	Polar & Charged Groups in Native Melittin	Polar & Charged Groups in CL	Bond Type
#1 −12.96 kJ/mol			Non-polar
#2 −12.12 kJ/mol	Gly12(O=CNH^δ+^_pb_) Arg22(CNH_2_^δ+^)	B-PO_4_^−^ II-C=O^δ−^	Polar-ionic Hydrogen
#3 −12.12 kJ/mol	1. Gln26(O=CNH_2_^δ+^) 2. Lys23(CN^+^H_3_) 3. Arg22(CNH_2_^δ+^) 3. Arg22(CNH_2_^δ+^) 3. Arg22(CNH_2_^δ+^) 4. Arg22(CNH^δ+^C) 5. Trp19(CNH^δ+^C) 5. Trp19(CNH^δ+^C)	a. A-PO_4_^−^ b. II-C=O^δ−^ c. B-CO^δ−^P a. A-PO_4_^−^ d. IV-CO^δ−^C a. A-PO_4_^−^ e. I-CO^δ−^C f. A-CO^δ−^P	Polar-ionic Polar-ionic Hydrogen Polar-ionic Hydrogen Polar-ionic Hydrogen Hydrogen
#4 −11.70 kJ/mol			Non-polar
#5 −11.70 kJ/mol	Lys23(CN^+^H_3_) Gln26(O=CNH_2_^δ+^) Arg22(CNH_2_^δ+^) Arg22(CNH^δ+^C) Arg22(CNH_2_^δ+^) Arg22(CNH_2_^δ+^) Lys23(CN^+^H_3_)	II-C=O^δ−^ A-PO_4_^−^ A-PO_4_^−^ A-PO_4_^−^ B-CO^δ−^P IV-CO^δ−^C II-C=O^δ−^	Polar-ionic Polar-ionic Polar-ionic Polar-ionic Hydrogen Hydrogen Polar-ionic
#6 −11.30 kJ/mol	Arg22(CNH_2_^δ+^)	II-C=O^δ−^	Hydrogen
#7 −11.30 kJ/mol	Lys21(CN^+^H_3_)	IV-C=O^δ−^	Polar-ionic
#8 −11.30 kJ/mol			Non-polar
#9 −11.30 kJ/mol	Gln26(O=CNH_2_^δ+^) Lys23(CN^+^H_3_) Trp19(CNH^δ+^C) Arg22(CNH_2_^δ+^) Arg22(CNH^δ+^C) Arg22(CNH_2_^δ+^) Arg22(CNH_2_^δ+^)	A-PO_4_^−^ II-C=O^δ−^ A-CO^δ−^P A-PO_4_^−^ A-PO_4_^−^ B-CO^δ−^P IV-CO^δ−^C	Polar-ionic Polar-ionic Hydrogen Polar-ionic Polar-ionic Hydrogen Hydrogen

**Figure 2:**
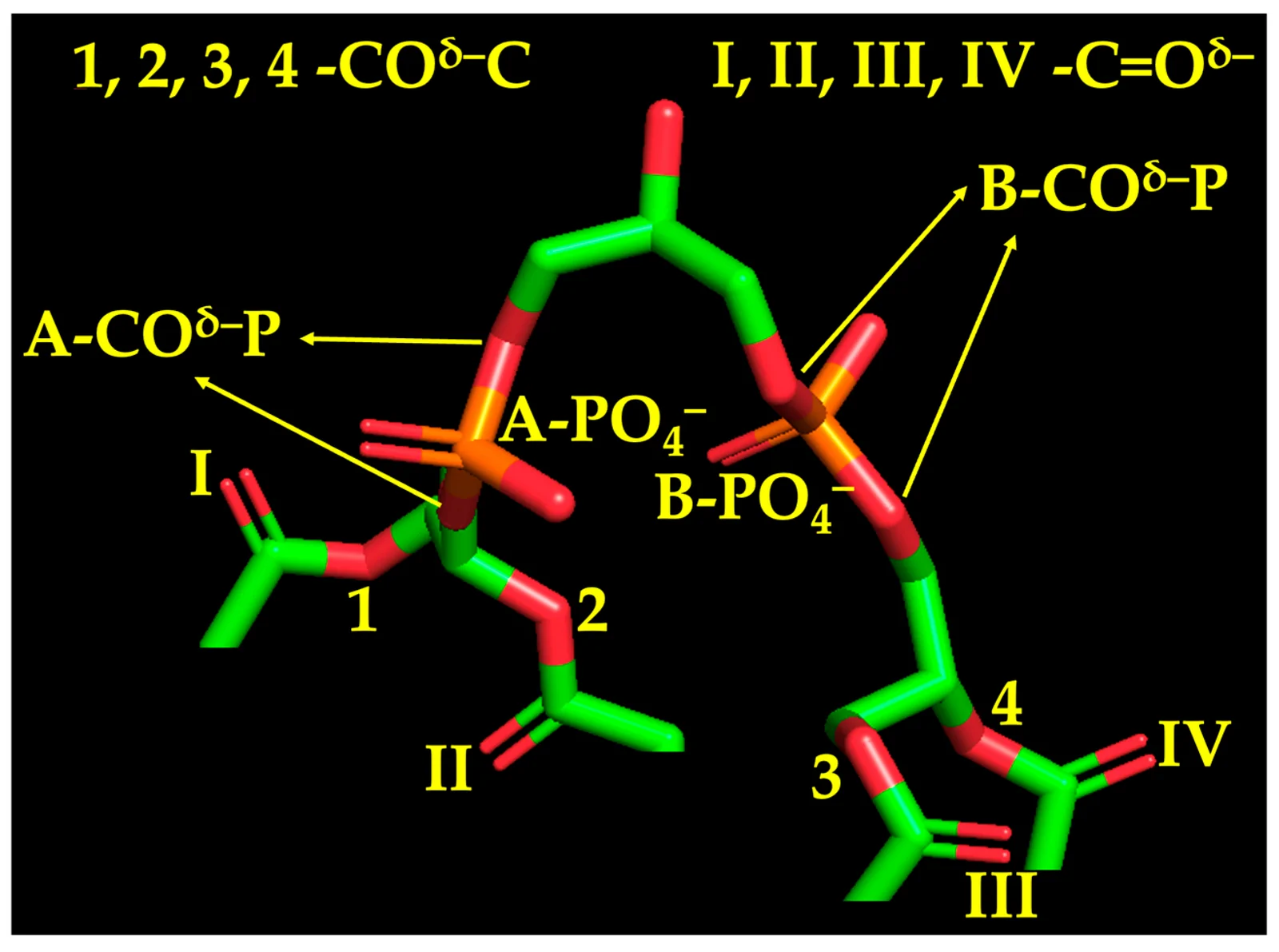
Notations for polar and charged groups in the polar head group of CL.

Arg22 is predicted by AutoDock simulations as the most abundant amino acid residue forming polar-ionic and hydrogen bonds in binding sites #2, #3, #5, #6, and #9, with the highest binding affinities observed in sites #2 and #3 (Table 1). Binding site #3 also features the greatest number of polar-ionic and hydrogen bonds, involving Trp19, Arg22, Lys23, and Gln26, with Arg22 contributing four out of the total eight intermolecular bonds (Table 1 and Figure 2). Of the six binding sites that involve polar-ionic and hydrogen bonds, Arg22 is absent from only one—binding site #7 (Table 1). This finding prompted us to generate a digital version of modified melittin in which Arg22 was replaced with glycine—an amino acid residue lacking an R-group side chain (Figure S1).

While AutoDock Vina predicted six amino acid residues in native melittin—Gly12, Trp19, Lys21, Arg22, Lys23, and Gln26—as forming polar-ionic and hydrogen bonds with the charged and polar moieties of CL, docking of the modified melittin (with Gly22 substituted for Arg22) predicted only four residues—Ser18, Trp19, Lys21, and Gln26—as forming ionic, polar-ionic and hydrogen bonds (Table 2). Figure S2 presents binding site #1 involving Ser18 and Trp19 in hydrogen bonding with phosphate and ester carbonyl groups of CL. As with native melittin, three binding sites (#6, #8, and #9) involving only non-polar bonding between the alkyl chains of CL and hydrophobic amino acid residues of the modified melittin were also predicted. Notably, binding affinity energies were lower at all binding sites for the modified melittin compared to the native protein (Table 2).

Thus, AutoDock Vina simulations predicted markedly different interaction patterns for native melittin and modified melittin (with Gly22 substituted for Arg22) with CL. This led us to hypothesize that the two melittins would induce different physiological outcomes in terms of modulating mitochondrial ATP synthase activity, affecting proton adsorption on the surface of CL-containing membranes, and triggering bilayer-to-non-bilayer lipid polymorphic transitions. To test this hypothesis, we produced two recombinant proteins in this work—native and modified melittins.

### 2.2. Purification of Fusion Proteins: Recombinant Native and Modified Melittin

**Figure 4:**
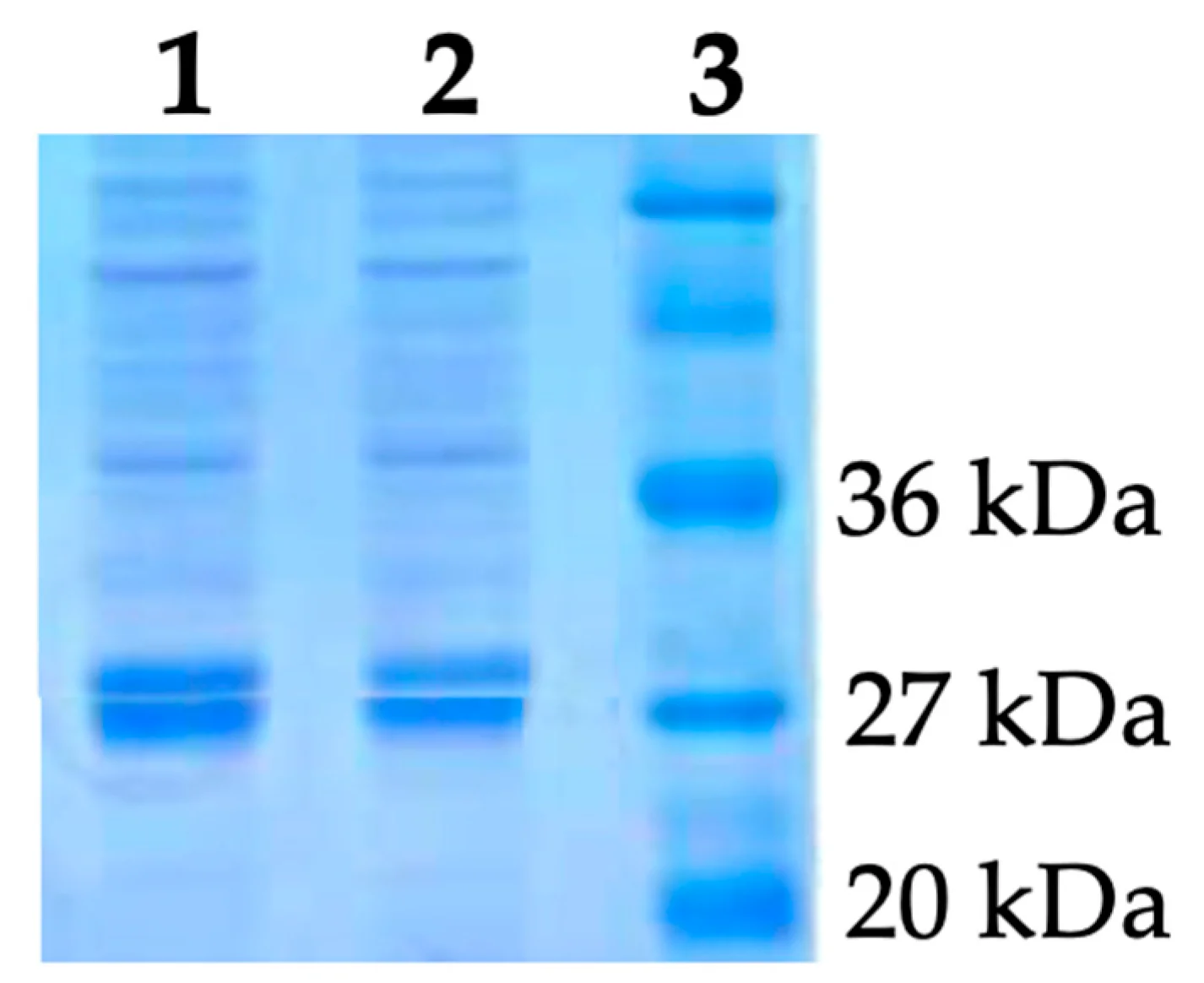
SDS-polyacrylamide (14%) gel electrophoresis of eluates from a glutathione Sepharose affinity column eluted with buffer B. Lane 1: eluate containing GST-native melittin; Lane 2: eluate containing GST-modified melittin; Lane 3: molecular weight markers from Takara (Dalian, China). This figure has been modified from the original gel image shown in Figure S3 for improved visualization.

The supernatants from the lysates of BL21(DE3) strains expressing either GST–melittin or modified GST–melittin were filtered through a 0.22 μm microporous membrane and subjected to high-performance affinity chromatography on a glutathione Sepharose column, as described in Section 5.5. After the column was washed with buffer A1, the tagged proteins—native GST–melittin or modified GST–melittin—were eluted with buffer B1 (see Section 5.5). Proteins in the eluates were dialyzed and lyophilized, and then analyzed by SDS-PAGE on a 14% gel (Figure 4).

As can be seen in Figure 4, both eluants display two prominent bands at approximately 27 kDa on the SDS-PAGE gel. These bands correspond to the molecular weights of pure GST (about 27 kDa) and the two fusion proteins—native and modified GST–melittins (about 30 kDa). This most likely indicates that expression of GST–melittin concurrently induces the overexpression of GST, as has been suggested in a study featuring melittin expression under similar conditions [41].

Proteins eluted from the glutathione Sepharose high-performance column—containing either native or modified GST–melittin fusion proteins—were separately treated with Prescission protease (Ppase) after which Ppase was precipitated as described in Section 5.6. The samples were then centrifuged and the supernatants, one from native and another from modified melittin digests, were dialyzed, lyophilized, and further fractionated by reverse-phase high-performance liquid chromatography on a Jupiter C5 column, as described in Section 5.6.

**Figure 5:**
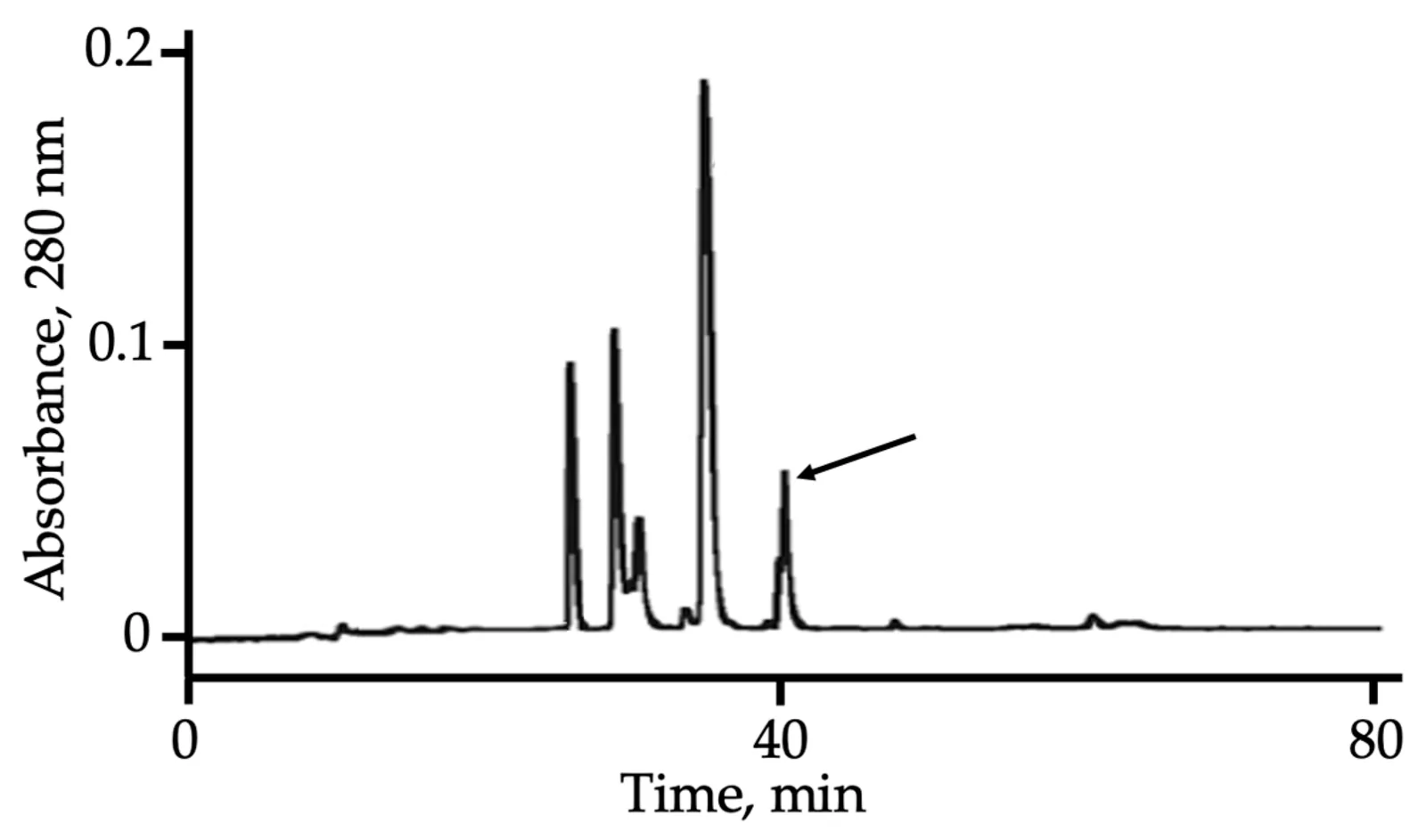
Reverse-phase (RP) chromatography on a Jupiter C5 column of the lyophilized supernatant of the digested mixture sample resulted from treating native GST–melittin with Ppase. A linear gradient of acetonitrile has been applied (see Section 5.6 for details). A fraction containing recombinant native or modified melittins indicated by arrow.

The chromatogram of the lyophilized digest mix containing native GST–melittin is shown in Figure 5. The chromatogram of the lyophilized digest mix with modified GST–melittin was virtually identical to that shown in Figure 4. In both cases, the recombinant native or modified melittin eluted in the final fraction at 40 min, as indicated by the arrow in Figure 5. After chromatography, the fractions were dialyzed and lyophilized prior to analysis by SDS-polyacrylamide (18%) gel electrophoresis.

**Figure 6:**
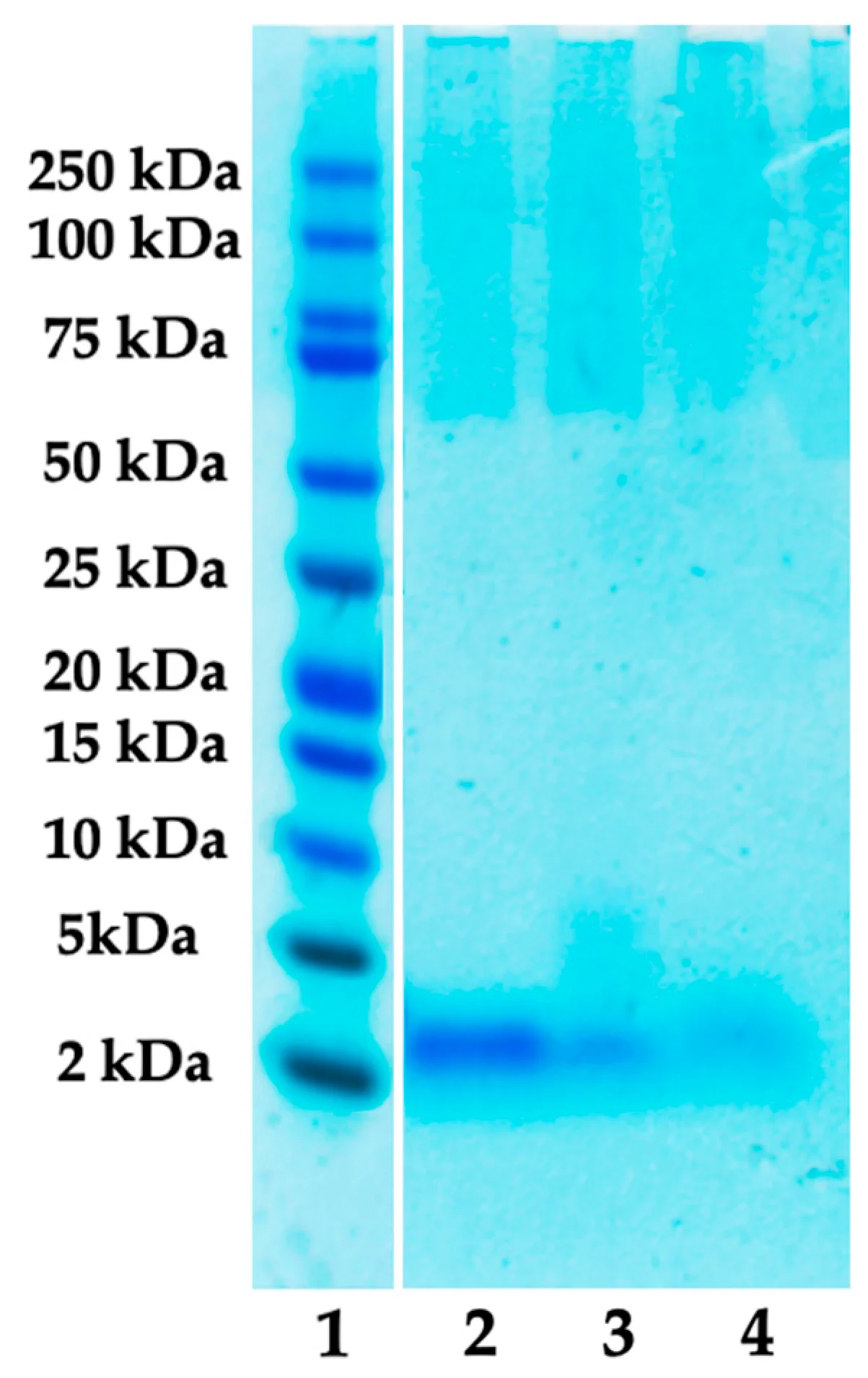
SDS-polyacrylamide (18%) gel electrophoresis of r-modified melittin (lane 2), r-native melittin (lane 3) and commercial melittin (98% purity) (lane 4). Lane 1: molecular weight markers from Presicion Plus Protein^TM^ Dual Xtra Standards (Bio-Rad, Carlsbad, CA, USA). This figure has been modified from the original gel image (shown in Figure S4) to reposition lane 1 next to the melittin lanes for better comparison.

The SDS-PAGE image (18% gel) shown in Figure 6 demonstrates that the final fraction from the reverse-phase chromatography represents homogeneous r-native and r-modified melittins, with molecular weights comparable to that of commercial melittin (Figure 6).

The positioning of the r-modified and r-native melittins alongside commercial melittin on the 18% SDS-PAGE gel shown in Figure 6 suggests that the additional N-terminal amino acid residues Gly–Pro–Leu–Gly–Ser, which contribute 0.411 kDa to the r-melittins and are encoded by the plasmid, do not visibly affect their migration through the 18% gel relative to commercial melittin.

### 2.3. Secondary Structure Analysis of r-Native Melittin and r-Modified Melittin by Circular Dichroism Spectroscopy

**Figure 7:**
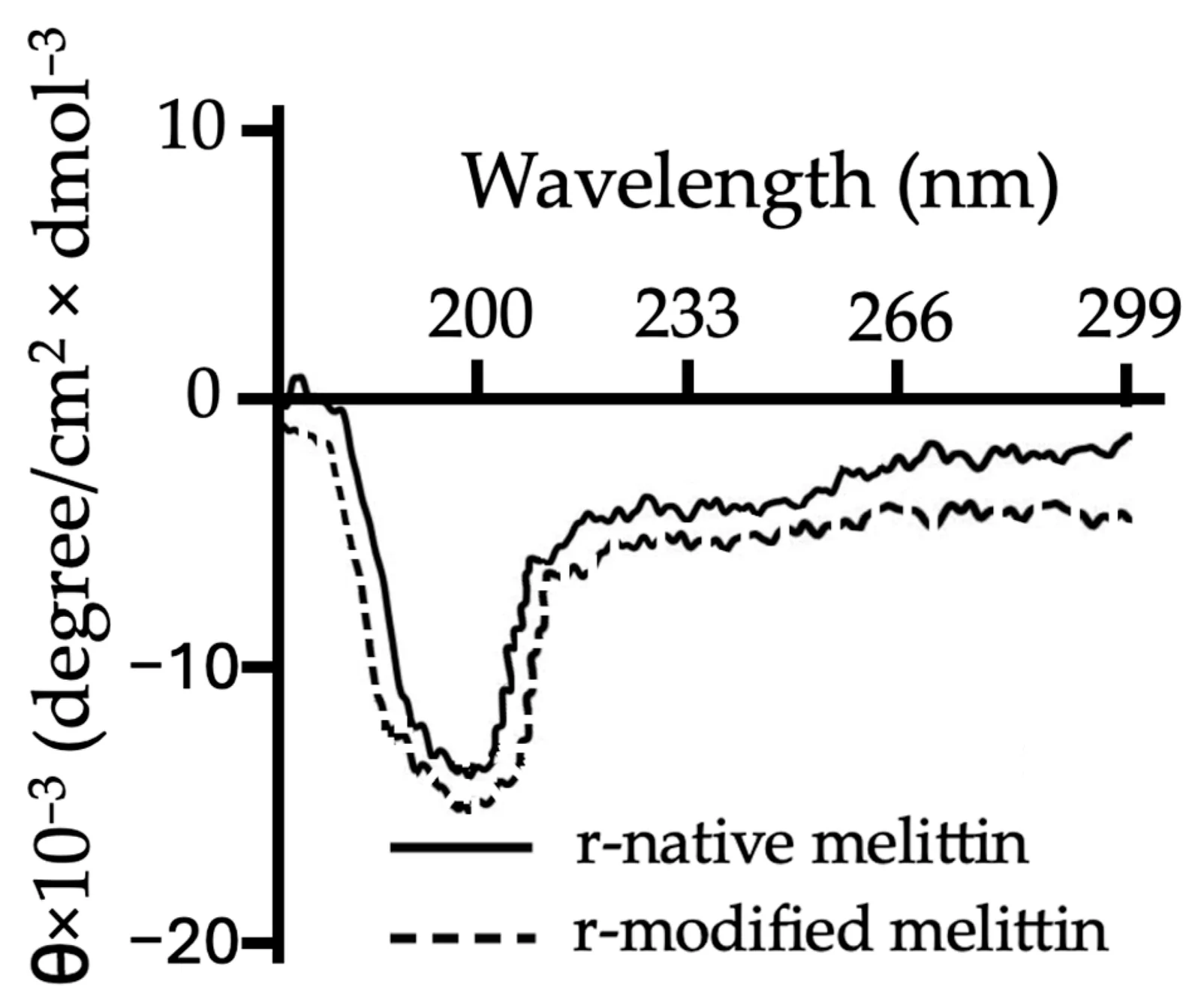
Circular dichroism spectra of r-native melittin (solid line) and r-modified melittin (dashed line) in phosphate-buffered saline.

The circular dichroism (CD) spectrum of r-native melittin (Figure 6) suggests a mixture of secondary structures, likely a combination of α-helical and unordered (random coil) conformations. The CD spectrum of commercial venom melittin was identical to that of r-native melittin, indicating that r-melittin and commercial venom melittin adopt identical conformations in an aqueous environment. The CD spectrum of r-modified melittin is very similar to that of r-native melittin and exhibits a similar overall trend, suggesting that the secondary structures of the two recombinant melittins are closely comparable. As shown in Figure 7, the residue ellipticities of recombinant native and modified melittins at 198 nm are 14.04 × 10^−4^ and 14.22 × 10^−4^ cm^2^/dmol, respectively.

### 2.4. Effects of r-Native Melittin and r-Modified Melittin on Proton Adsorption by CL Containing Phospholipid Membranes

In recent years, the adsorption of protons onto the surface of phospholipid membranes has attracted considerable research attention, as this phenomenon has novel potential implications in bioenergetics. Phospholipid membranes have been viewed as proton capacitors, storing energy to fuel the ATP synthase machinery [35,36,42]. The two phosphate moieties of cardiolipin (CL) are particularly effective in acting as proton capacitors. They do not trap protons in the sense of classic ionic bonds; rather, they draw protons out of the bulk water through long-range electrostatic forces, functioning like a negatively charged sponge that holds protons at the membrane interface in a dynamic exchange with hydroxonium ions. It is believed that this loose, reversible electrostatic capture is how the IMM—rich in CL—concentrates protons at the membrane surface to drive ATP synthesis [42,43].

We have recently demonstrated that anionic proteins from bee venom [33], as well as cationic and anionic proteins from *Latrodectus pallidus* spider venom, can increase proton adsorption onto model membranes composed of zwitterionic phosphatidylcholine (PC), phosphatidylethanolamine (PE), and sphingomyelin (SM), as well as acidic phosphatidylserine (PS) and phosphatidylinositol (PI) [34]. In the present study, we used a model membrane mimicking the phospholipid composition of the IMM, consisting of 80 mol% PC and 20 mol% CL. In this membrane, only CL—not PC—can adsorb protons at pH 6.88, which corresponds to the pH detected in the mitochondrial intermembrane space [44].

As shown in our computer simulations described above, Arg22 of cationic melittin forms polar-ionic and hydrogen bonds with both phosphate groups of CL, thereby rendering CL incapable of adsorbing protons from the bulk water. To investigate the role of Arg22 in modulating proton adsorption by PC + CL membranes, we produced two recombinant melittins: one with the native amino acid sequence and another with Gly substituted for Arg22.

We measured proton adsorption onto the surface of liposomal PC + CL membranes by monitoring the pH change in saline prepared at pH 6.88 upon addition of liposomes. The concentration of adsorbed H^+^ was determined by subtracting the [H^+^] in the liposome-containing saline solution from that in pure saline solution.

As shown in Table 3, r-native melittin caused only a negligible increase in proton adsorption when its concentration was raised from 6.00 × 10^−11^ M to 2.40 × 10^−10^ M. However, further increases in r-native melittin concentration resulted in a sharp rise in proton adsorption on the membrane surface. The effect of commercial bee venom melittin on the same liposomes was identical (Table S1). This observation suggests that r-native melittin and commercial bee venom melittin are structurally virtually identical.

**Figure 8:**
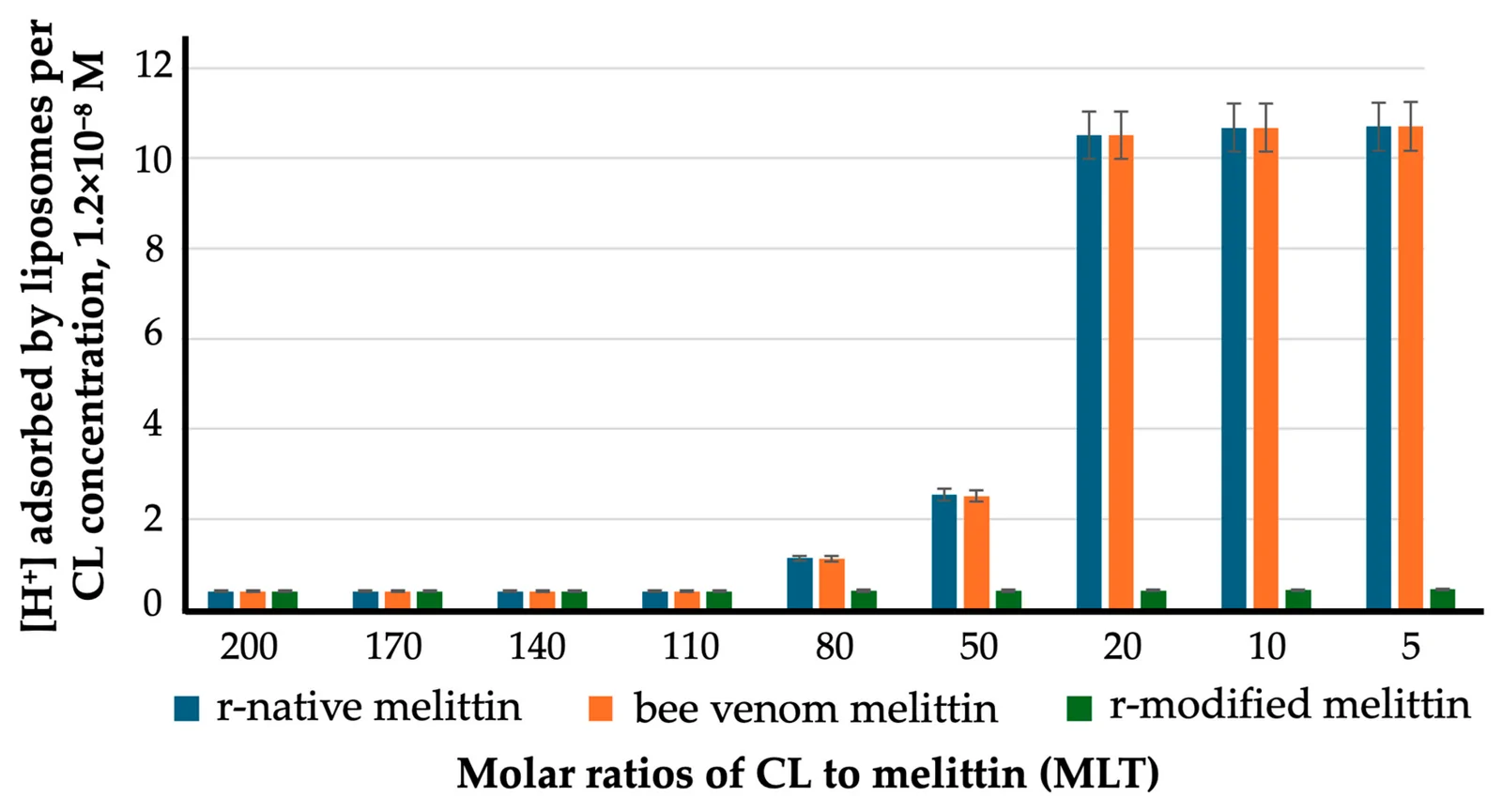
Proton adsorption by liposomes given as a concentration of adsorbed protons per CL concentration as a function of increase in melittin concentration. The liposome samples for each reading taken at 25 °C were prepared in triplicate. The error bars represent standard deviation between three readings for each data point which never exceeded 5%. ANOVA with Tukey’s test was used to compare means between groups treated with different melittins and Student’s *t*-test was used to compare means for the groups treated with the same type of melittin. A *p*-value < 0.05 was considered statistically significant.

Notably, the r-modified melittin—with glycine substituted for arginine at position 22—had only a negligible effect on proton adsorption onto the membrane surface, even at high concentrations (Table 4). As clearly shown in Figure 8, the effects of r-native melittin and commercial bee venom melittin were indistinguishable, whereas the effect of the r-modified melittin was markedly different and barely observable. This observation strongly suggests that Arg22 plays a crucial role in modifying the surface of the PC + CL membrane in a manner that enhances proton adsorption.

Notably, our statistical analysis—using Student’s *t*-test to assess changes in proton adsorption within groups and ANOVA with Tukey’s test to assess changes between groups—consistently produced *p*-values below 0.05. This confirms that changes in proton adsorption were caused by treatment with increasing concentrations of either r-native melittin or commercial bee venom melittin.

### 2.5. Effects of r-Native Melittin and r-Modified Melittin on ATP Production by Mitochondrial ATP Synthase

From the very beginning of the chemiosmotic theory of mitochondrial bioenergetics [45], researchers have noted the phenomenon of proton adsorption onto membrane surfaces, and it has become increasingly evident that membrane-localized protons couple oxidative phosphorylation [46,47,48,49]. Meta-stable bonds between protons and membrane surfaces have been reported [50], and it has been shown that the mitochondrial membrane adsorbs protons acting as a conjugate base, while serving as a Brønsted acid when supplying protons as a substrate for ATP synthase [51].

A recent review article [52] underscored the importance of localized protons in driving conformational shifts within the F_1_ subunit and initiating the phosphorylation reaction (ADP → ATP). It has been proposed that protons moving through the F_o_ subunit do not dissipate into the surrounding water; instead, they establish a pH gradient between F_o_ and F_1_, which facilitates proton delivery to F_1_. Mechanistically, as protons traverse the F_o_ subunit, they attach to carboxyl groups on amino acid residues inside F_1_, neutralizing electrostatic interactions between charged residues of the rotor and stator, thereby inducing conformational changes in the enzyme [52]. Protonation occurs at the onset of ATP synthesis and during phosphorylation, whereas deprotonation halts F_o_ rotation, suppressing both ATP production and hydrolysis. Overall, the review highlighted several essential functions of protons, including their role in coupling within ATP synthase, mediating charge and energy transfer, enabling rotational movement through conformational adjustments, and directly participating in the ATP synthesis reaction [52].

In our recent work, we demonstrated that acidic proteins from bee venom amplify proton adsorption onto phospholipid membrane surfaces, suggesting that protein–lipid complexes on the membrane may act as proton capacitors, potentially storing energy for driving ATP synthesis [33]. We have also shown that basic and acidic proteins from *Latrodectus pallidus* spider venom, whose amino acid sequences are highly homologous to human myelin basic protein isoforms, can adsorb protons onto membrane surfaces and trigger the formation of non-bilayer lipid structures [34]. In our earlier studies, we showed that basic membrane-active proteins—cytotoxins from cobra venom and melittin from bee venom—can enhance mitochondrial ATP synthase activity by inducing non-bilayer phospholipid structures in the inner mitochondrial membrane [2,17,23,26,30,31,32].

In the present work, we demonstrated that r-native melittin significantly increases proton adsorption on the surface of CL-containing membranes, whereas r-modified melittin—with glycine substituted for arginine at position 22—had only a negligible effect. This raises the question of whether r-native and r-modified melittins differentially affect mitochondrial ATP synthase activity and the induction of non-bilayer phospholipid structures.

**Figure 9:**
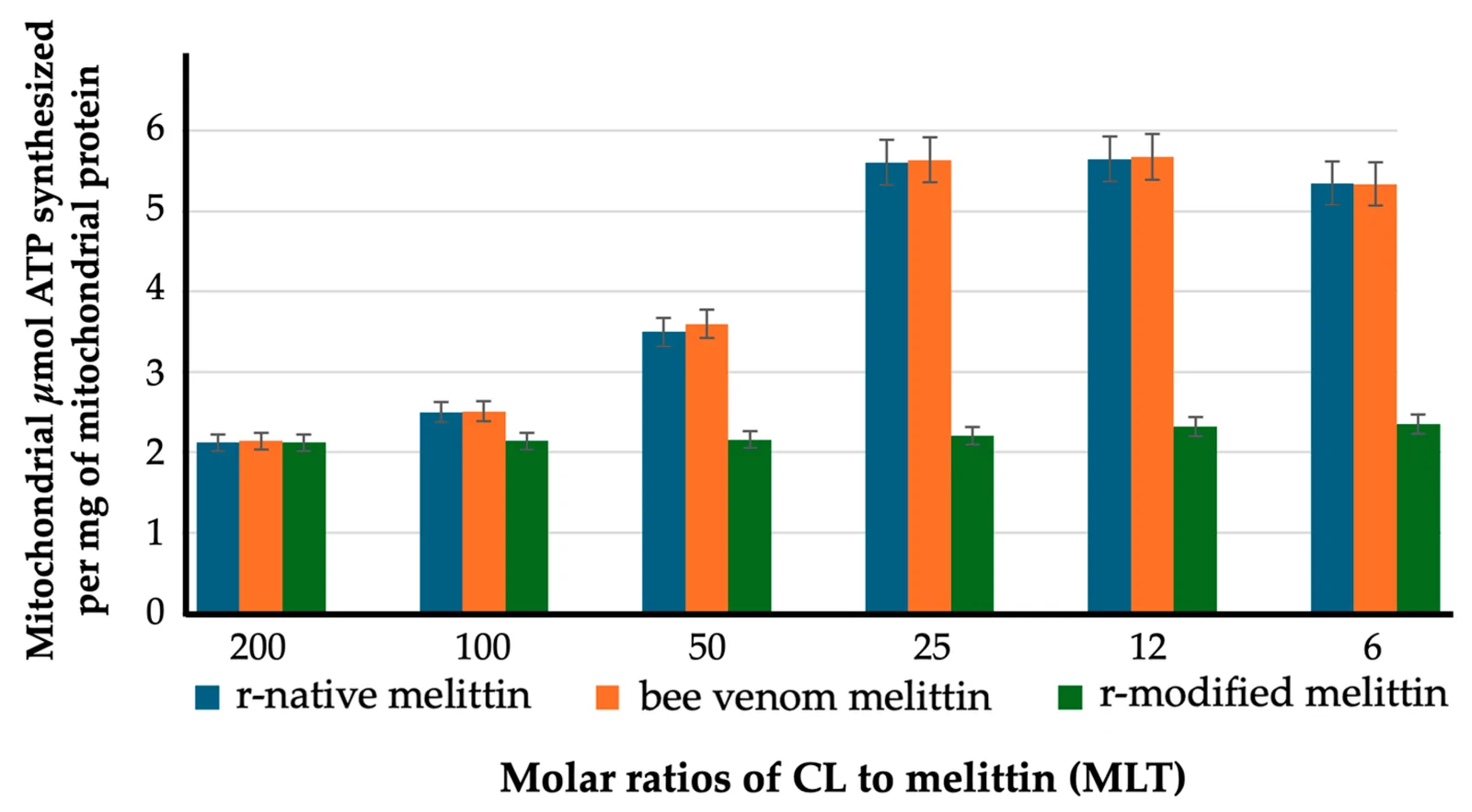
Mitochondrial ATP synthase activity as a function of increasing melittin concentration. The phospholipid concentration in each mitochondrial sample was estimated to be 3.2 × 10^−2^ M, which corresponds to approximately 0.64 × 10^−2^ M CL. ATP synthase activity was measured at 37 °C in the presence of 1.5 mM succinate. Measurements were performed in a control sample (no melittin) and in samples treated with r-native melittin, commercial bee venom melittin, or r-modified melittin at concentrations of 3.20 × 10^−5^ M, 6.40 × 10^−5^ M, 1.28 × 10^−4^ M, 2.56 × 10^−4^ M, 5.33 × 10^−4^ M, or 1.07 × 10^−3^ M. These concentrations correspond to CL-to-melittin molar ratios of 200, 100, 50, 25, 12, and 6, respectively. ATP synthase activity for each mitochondrial sample was calculated from triplicate measurements. All samples were prepared from a single stock of isolated bovine heart mitochondria. Inter-sample standard deviation was less than ±4%, and inter-experimental variability remained below ±5%. An ANOVA with Tukey’s test was used to compare means between groups treated with different melittins, and Student’s *t* test (two-tailed) was used to compare means within groups treated with the same melittin. A *p*-value < 0.05 was considered statistically significant.

In this study the ATP level measured at 37 °C in a control bovine heart mitochondrial sample was 2.12 µmol of ATP synthesized per mg of mitochondrial proteins. Treating mitochondria with 3.2 × 10^−5^ M of r-melittin, bee venom melittin, or r-modified melittin at a CL-to-melittin molar ratio of 200 did not affect mitochondrial ATP level (Figure 9). However, with an increase in concentration of r-melittin or bee venom melittin in mitochondrial samples the ATP level gradually increased, reaching a maximum of 5.65 µmol per mg of mitochondrial proteins at a CL-to-melittin molar ratio of 12. However, at a CL-to-melittin molar ratio of 6 the ATP level slightly declined to 5.35 µmol per mg of mitochondrial proteins which has probably resulted from a slight perturbation in mitochondrial membrane structure caused by the increased concentration of r-native melittin or bee venom melittin. Notably, r-modified melittin did not affect mitochondrial ATP synthase activity (Figure 9), which strongly suggests that Arg22 plays an important role not only in adsorption of protons on membrane surface, but also in facilitating mitochondrial ATP synthase activity.

Notably, low *p*-values (always below 0.05) from Student’s *t* test and ANOVA with Tukey’s test confirm that changes in mitochondrial ATP synthase activity within groups and between groups treated with increasing concentrations of either r-native melittin or commercial bee venom melittin are caused by the actions of melittins.

### 2.6. Effects of r-Native, Commercial and r-Modified Melittin on Polymorphic Lipid Phase Transition in CL-Containing Phospholipid Membranes

So far, we have observed that r-native melittin and commercial bee venom melittin enhance proton adsorption by CL-containing membranes and induce an increase in mitochondrial ATP production, whereas r-modified melittin—with glycine substituted for arginine at position 22—lacks these abilities. In this section, we investigate whether these three melittins differ in their capacity to affect phospholipid packing in CL-containing membranes and to induce the formation of non-bilayer structures.

Previously, we have used ^1^H-NMR spectroscopy of sonicated PC liposomes enriched with CL to study the formation of non-bilayer structures in liposomal membranes induced by cobra venom cytotoxic polypeptides [26,30,31,32]. The ^1^H-NMR signal from the hydrogens of the trimethylammonium N^+^(CH_3_)_3_ group of PC molecules is well separated from signals of other lipid hydrogens in the ^1^H-NMR resonance frequency region (expressed in ppm). This yields a well-defined single ^1^H-NMR signal that unambiguously identifies PC molecules in sonicated liposomes.

**Figure 10:**
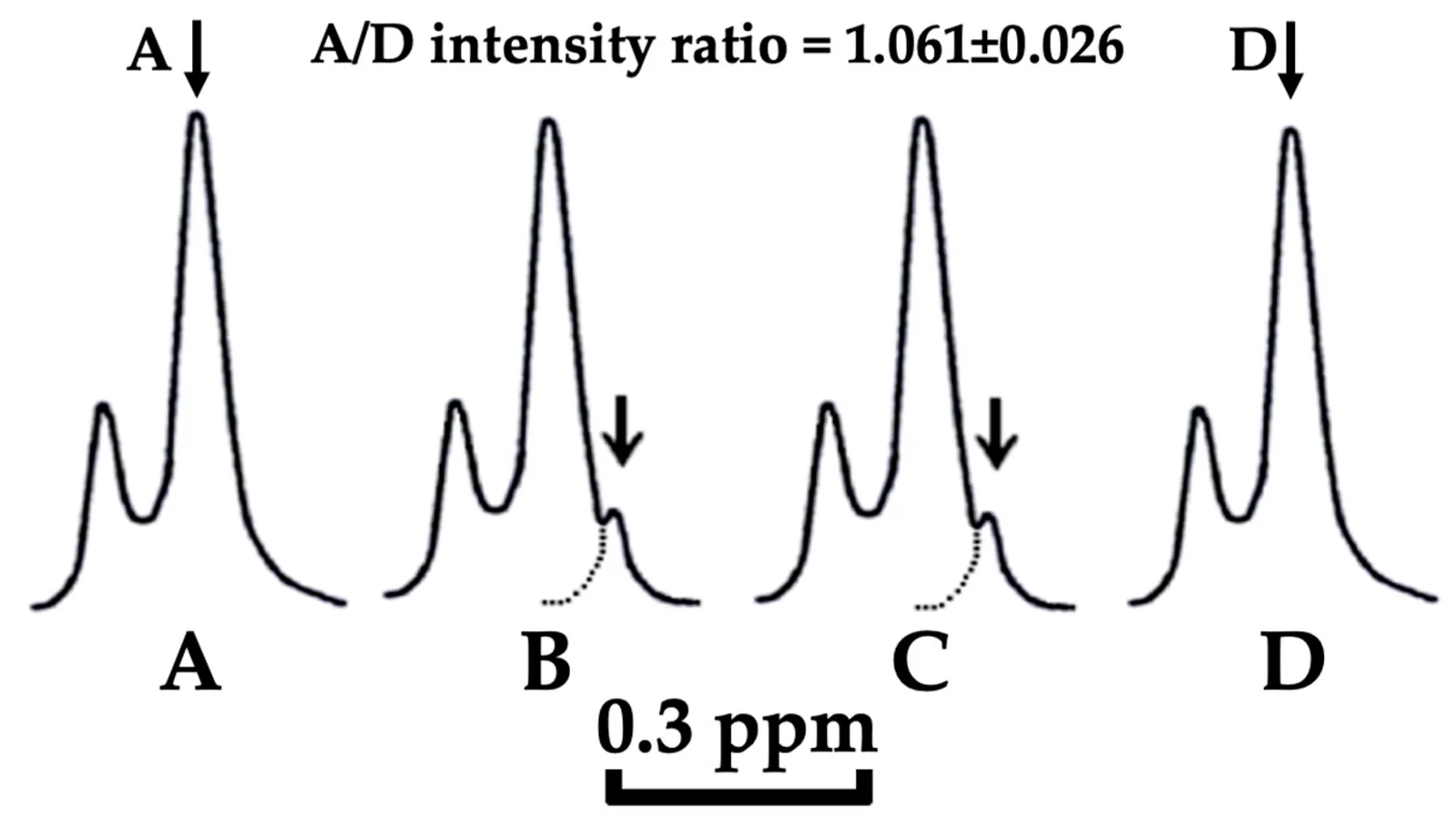
^1^H-NMR spectra from the N^+^(CH_3_)_3_ groups of PC in unilamellar liposomes composed of PC + 20 mol% CL in absence of melittin (**A**), treated with r-native melittin (**B**), commercial bee venom melittin (**C**), or r-modified melittin (**D**). Total lipid concentration was 1.5 × 10^−2^ M, and melittin concentration was 1.2 × 10^−4^ M (corresponding to a CL-to-melittin molar ratio of 25). All measurements were performed in the presence of K_3_[Fe(CN)_6_] (10 µL of saturated K_3_[Fe(CN)_6_] solution per 1 mL of liposome suspension). Liposome samples were first incubated for 15 min at 25 °C prior to the addition of K_3_[Fe(CN)_6_], followed by a further 10-min incubation before the addition of melittin (r-native, commercial, or r-modified), and ^1^H-NMR spectra were recorded 5 min after that. Arrows in spectra (**B**,**C**) indicate the presence of non-bilayer structures. The spectra shown are representative of three independent experiments, which yielded nearly identical results.

Upon the addition of ferricyanide [Fe(CN)_6_]^3−^ ions to a suspension of PC-containing liposomes, the single signal from the N^+^(CH_3_)_3_ group of PC splits into a doublet of uneven intensities. This occurs because the Fe(CN)_6_^3−^ ions interact with the N^+^(CH_3_)_3_ groups of PC in the outer leaflet of the liposome, shifting their signal toward a higher resonance field, while the signal from the N^+^(CH_3_)_3_ groups in the inner leaflet remains unchanged. The unequal intensities of the doublet signals arise from the larger surface area of the outer leaflet compared to the inner leaflet. The smaller the liposomes, the greater the difference in signal intensities between the two leaflets. Sonicated liposomes are small, which is why the signal intensity from the outer leaflet is significantly higher than that from the inner leaflet as shown in ^1^H-NMR spectrum of control PC + CL liposomes not treated with melittin (Figure 10A).

As shown in Figure 10B,C the two ^1^H-NMR spectra from PC + CL liposome samples—one treated with r-native melittin and the other with commercial bee venom melittin at a CL-to-melittin molar ratio of 25—display, in addition to the doublet signals, a third signal on the high-field side of the outer leaflet signal (indicated by arrows in spectra B and C). As described in our previous studies [23,25,26,30,31,32,53,54], this third signal originates from PC molecules in non-bilayer structures with high surface curvature. Such curvature allows closer interaction of the N^+^(CH_3_)_3_ groups on the outer leaflet with Fe(CN)_6_^3−^ ions, resulting in a further shift in their resonance frequency toward higher field in the spectrum. These non-bilayer structures, although predominantly composed of CL, also include PC molecules; however, CL molecules—which lack N^+^(CH_3_)_3_ groups—do not produce ^1^H-NMR signals and therefore are not directly observed in these spectra [26,30,32,53,54].

It should be noted that PC + CL liposomes treated with r-modified melittin at a CL-to-melittin molar ratio of 25 did not trigger the formation of non-bilayer structures, as shown in the ^1^H-NMR spectrum in Figure 10D. In contrast to spectra B and C—which correspond to liposomes treated with r-native melittin or commercial bee venom melittin—spectrum D lacks the third high-field signal. Additionally, the intensity of the outer leaflet signal in spectrum D is slightly lower than that in spectrum A (signal A-to-signal D intensity ratio of 1.061 ± 0.026), which is likely due to the binding of r-modified melittin to the surface of PC + CL liposomes without inducing non-bilayer structures. Although this observation provides indirect evidence of r-modified melittin interaction with membrane, it is not a direct measurement of binding affinity.

Notably, treatment of PC + CL liposomes with 3.0 × 10^−5^ M of r-native melittin, r-modified melittin, or bee venom melittin, corresponding to a CL-to-melittin molar ratio of 100 at which no increase in proton adsorption or ATP production was observed, did not generate a ‘high-field non-bilayer signal’. This finding strongly suggests that non-bilayer structures facilitate an increase in membrane proton adsorption and mitochondrial ATP production.

These findings, together with the results presented in Section 2.4 and Section 2.5, lead to the conclusion that Arg22 plays a crucial role in the interaction of melittin with CL-containing membranes, resulting in proton adsorption on the membrane surface, formation of non-bilayer structures, and enhancement of mitochondrial ATP synthase activity.

## 3. Discussion

In this study, we have established for the first time that native melittin enhances proton adsorption on the surface of PC + 20 mol% CL membranes. We have also extended our previous finding that melittin disturbs the packing of phospholipid membranes enriched with anionic phospholipids [2], and now demonstrate that melittin triggers the formation of non-bilayer structures. Additionally, we have expanded on our earlier work showing that melittin synergistically enhances mitochondrial ATP synthase activity in the presence of alcohols [23], and now show that melittin alone is sufficient to enhance this activity. In this section, we discuss the mechanistic basis by which melittin affects proton adsorption on the surface of PC + 20 mol% CL membranes, how this relates to the induction of non-bilayer structures, and how these events collectively lead to the enhancement of mitochondrial ATP synthase activity.

In PC + CL membranes, PC is unlikely to adsorb protons, as the negatively charged phosphate group of PC is balanced by its positively charged trimethylammonium group, resulting in a zeta potential close to zero at neutral pH in saline [55,56]. CL, on the other hand, possesses two phosphate groups that are fully deprotonated at pH above 4, giving CL two negative charges on the surface of phospholipid liposomes at physiological pH [57]. This highly negative surface potential creates an electrostatic gradient on the membrane surface that attracts protons from the bulk water via long-range electrostatic forces, holding them loosely at the interface in a dynamic exchange. This interaction is physical rather than chemical [57,58]. The protons do not “bind” to the phosphate groups through permanent chemical bonds; instead, they are drawn to the negatively charged surface by electrostatic forces, forming a dynamic “proton cloud.” In this cloud, protons attracted to the surface exchange with those in the bulk water on a nanosecond timescale and then return. This reversible electrostatic capture of protons on the membrane surface is crucial for driving mitochondrial and extramitochondrial ATP synthesis [36,42]. Notably, other polar groups of cardiolipin—such as C–O–C (ether), C=O (ester carbonyl), and C–OH (hydroxyl)—do not adsorb protons at physiological pH.

The proton-adsorbing capacity of a phospholipid membrane enriched with a membrane-active protein can be estimated by counting the number of charged and polar moieties of phospholipids and amino acid residues capable of adsorbing protons. Additionally, intermolecular bonds between the charged and polar moieties of phospholipids and those of amino acid residues should be analyzed with particular focus on the moieties capable of proton adsorption, as the formation of an intermolecular bond effectively neutralizes their proton-adsorbing capacity [59].

Of the 26 amino acid residues in native melittin, 21 residues lack proton-adsorbing capability. The five basic residues are two arginine residues (Arg22 and Arg24) and three lysine residues (Lys7, Lys21, and Lys23). Each of these basic residues adsorbs one proton at physiological pH through the formation of a covalent coordinate bond between the nitrogen of the R-side chain and a proton. However, these basic residues will not adsorb protons from the bulk water in our experimental setting as these residues are already protonated at pH 6.88.

In the present work, AutoDock simulations of CL interaction with the molecular surface of native melittin produced nine binding sites with the highest binding affinities. Three of these sites result solely from London dispersion forces between the alkyl chains of CL and hydrophobic amino acid residues on the surface of native melittin (Table 1, binding sites #1, #4, and #8). The remaining binding sites feature polar-ionic and hydrogen bonds involving the two phosphate groups of CL and residues Lys21, Lys23, and Arg22 of melittin. Notably, Lys7 and Arg24 were not involved in bonding.

Why do Lys7 and Arg24 not form bonds with the charged and polar groups of CL? The most likely reason is their location on the molecular surface of melittin. Lys7 resides in the center of a hydrophobic stretch, making it poorly accessible for ionic, polar-ionic, or hydrogen bonding with the CL polar head. Meanwhile, Arg24 appears to be shielded from such interactions by the amide groups of the side chains of Gln25 and Gln26.

Thus, the interaction of melittin with CL effectively “discharges” the proton-adsorbing ability of the two phosphate groups of CL. Hence, in theory, melittin should decrease proton adsorbing capacity of PC + CL membrane. However, due to the low concentration of melittin used in this study, it is not likely to markedly affect the overall proton-adsorbing capacity of the CL-containing membrane. The reduction in the extent of the “proton cloud” caused by melittin should probably be negligent in our experimental setting.

AutoDock simulations of CL interaction with the modified melittin (with Gly22 substituted for Arg22) also predicted nine binding sites with the highest binding affinities. The three binding sites on the molecular surface of the modified melittin involved only London dispersion forces between the CL alkyl chains and hydrophobic amino acid residues (Table 2, binding sites #6, #8, and #9). In the remaining six binding sites, attraction between CL and modified melittin involved ionic, polar-ionic, and hydrogen bonds. With respect to the proton-adsorbing moieties of CL, the intermolecular bonds in binding sites #3 and #7 involved the two phosphate groups of CL which formed a polar ionic bond with Gln26, a hydrogen bond with Ser18, and an ionic bond with Lys21 (Table 2). This observation means that each modified melittin molecule could fully neutralize the proton-adsorbing capacity of one CL molecule. However, given the low concentrations of melittin used in this study, modified melittin should not, in theory, significantly affect the overall proton-adsorbing capacity of the CL-containing membrane.

Our study on proton adsorption capacity of PC + CL liposomes consistently showed a molar ratio of adsorbed protons to CL of 0.4 for liposomes free of melittin (Table 3, Table 4 and Table S1), suggesting that, on average, 2.5 CL molecules are required to adsorb one proton in PC + CL membranes. This indicates that it is not particularly easy for the phosphate groups of CL to sequester protons from hydroxonium ions in the bulk water. Notably, treatment of PC + CL liposomes with r-modified melittin aligned well with our AutoDock simulation results, as r-modified melittin caused no visible change in proton adsorption across the entire range of CL-to-melittin molar ratios tested (from 200 to 5) (Table 4 and Figure 8).

In contrast, treatment with r-native melittin showed no visible effect on proton adsorption only at CL/melittin molar ratios ranging from 200 to 110, where the adsorbed proton-to-CL molar ratio remained at 0.4. However, at CL/melittin molar ratios ranging from 80 to 5, proton adsorption increased exponentially (Table 3 and Figure 8). The effect of commercial bee venom melittin on proton adsorption in PC + CL liposomes was identical to that of r-native melittin (Table S1 and Figure 8). These results do not align with the predictions of the AutoDock simulations, which suggested that one native melittin molecule can neutralize the proton-adsorbing ability of the two phosphate groups of a CL molecule.

As shown in Table 3 and Table S1, at a CL-to-native (or bee venom) melittin molar ratio of 20, one CL molecule adsorbs an average of 10.5 protons from the bulk water. Our AutoDock simulations suggest that, under these conditions, the single native melittin should neutralize proton adsorbing capacity of one CL molecule, while the remaining 19 CL molecules—free of melittin—should adsorb 7.6 protons collectively (since, according to our proton adsorption study, one CL molecule in a melittin-free PC + CL membrane adsorbs 0.4 protons). Thus, theoretically, 20 CL molecules with one native melittin should adsorb 7.6 protons in total, corresponding to an average of 0.38 protons per CL. However, our experimental data shows 10.5 protons adsorbed per CL at this molar ratio.

Similarly, at a CL-to-native melittin molar ratio of 5, the theoretical calculation gives 0.32 protons adsorbed per CL molecule, whereas Table 3 and Table S1 show 10.7 protons per CL molecule. This substantial discrepancy between theoretical predictions and experimental observations suggests that, starting at a CL-to-native melittin molar ratio of 20 and above, the PC + CL bilayer membrane undergoes a structural transition that enables it to entrap significantly more protons than the bilayer membrane alone.

Notably, the results of our present work on ATP synthase activity in mitochondrial samples treated with r-native melittin, commercial bee venom melittin, or r-modified melittin align very well with our studies on proton adsorption by PC + CL liposomal membranes treated with the same melittin samples (Figure 9). Treatment of mitochondria with r-modified melittin did not visibly affect ATP synthase activity. In contrast, treatment with r-native or commercial bee venom melittin triggered a sharp increase in ATP synthesis at CL-to-melittin molar ratios ranging from 50 to 6. This pattern of ATP synthase activity closely resembles the proton adsorption pattern shown in Figure 8. The close alignment in the activities of the three different melittin samples—both in triggering proton adsorption by liposomes and in enhancing ATP synthesis by mitochondria—suggests exitance of a functional link between proton accumulation on the membrane surface and mitochondrial ATP synthesis.

In this work, we have also shown that treatment of PC + CL liposomes with r-native or commercial bee venom melittin at a CL-to-melittin molar ratio of 25—a ratio at which these melittins induce a sharp increase in both membrane proton adsorption and mitochondrial ATP synthase activity—leads to the formation of non-bilayer lipid structures (Figure 10). Importantly, the r-modified melittin, which contains Gly22 instead of Arg22, does not enhance proton adsorption, does not induce non-bilayer lipid structures in PC + CL liposomes, and does not increase mitochondrial ATP synthase activity. These findings strongly suggest that Arg22 plays a critical role in linking these events together.

In our previous work, we proposed—based on extensive experimental and computational data—a mechanism for the formation of non-bilayer lipid structures in CL-containing membranes induced by interaction with small cationic proteins [2,26,30,31,32]. The core concept underlying the transformation from bilayer to non-bilayer phospholipid packing centers on the electrostatic interaction between cationic proteins and anionic CL molecules, which reduces the effective size of the CL polar head and triggers adaptation to non-bilayer packing [26,30].

It has been hypothesized that one of the roles of non-bilayer structures in the IMM is to compartmentalize the intra-crista space through non-bilayer transitions in IMM regions of maximum curvature, protruding from rafts on opposing cristae membranes. This compartmentalization increases proton concentration near the F_o_ subunit of ATP synthase dimers, thereby promoting ATP synthesis [31,60].

**Figure 11:**
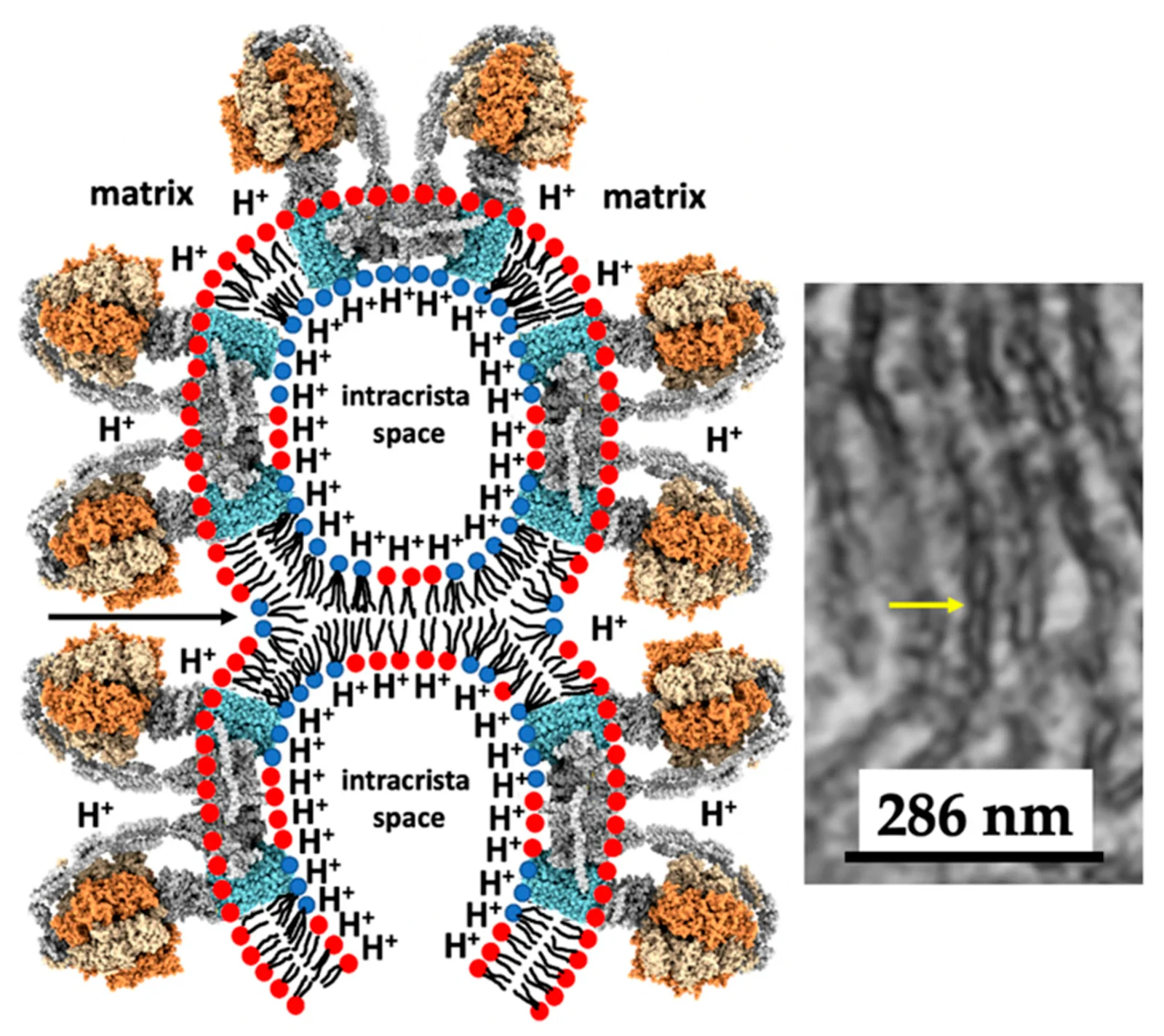
Compartmentalization via intermembrane junctions enhances proton concentration on the inner membrane surface of cristae. The (**left**) panel presents a schematic depiction of two crista compartments forming rafts on their outer surfaces, which are separated by an intermembrane junction (black arrow). The atomic model of ATP synthase (PDB # 6B8H) was generated by Dr. Semen V. Nesterov of the Moscow Institute of Physics and Technology using UCSF ChimeraX [64]. In this model, the α- and *β*-subunits of the catalytic domain are colored yellow and orange, respectively; the C-ring is shown in blue, while all other subunits appear in gray. CL polar heads are depicted in blue, and the polar heads of other IMM phospholipids with two alkyl chains are shown in red. For visual clarity, the alkyl chains of phospholipids have been omitted from regions overlaying the F_o_ subunits of ATP synthase. The (**right**) panel shows a transmission electron micrograph highlighting intermembrane junctions between cristae membranes (yellow arrow), which demarcate individual cristae rafts. This figure is adapted from [63].

Based on our latest experimental and computational data, this compartmentalization hypothesis has recently been refined. It now proposes that protons are not delocalized in bulk water but are instead localized on the membrane surface, thereby concentrating more protons at the F_o_ units of ATP synthase dimers (Figure 11). Protons migrate to regions of highest surface curvature on the inner cristae membranes—not only at the cristae apex but also at the peaks of lipid rafts—with each ATP synthase of the dimer positioned on opposite sides of the apex or raft peak [26,30,43,59,61,62,63].

A cationic protein—such as cobra venom cytotoxin—serves as the initiation site for contact between membranes, promoting bilayer-to-non-bilayer transitions that lead to the formation of an inverted micelle. This micelle subsequently evolves into an intermembrane junction within the intra-cristae space, linking opposing cristae membranes to form a compartment [30,61]. Naturally occurring cationic proteins in the cristae and intra-cristae space—including cytochrome c, creatine kinase, and misfolded c-ring subunits not integrated into ATP synthase—can similarly induce these junctions [30,59,61]. These intermembrane junctions are transient, appearing and dissolving in response to fluctuations in proton density along the inner membrane surface of the cristae [30,59]. Because of their short-lived nature, they do not hinder the movement of biomolecules through the bulk solution of the intra-cristae space [30,59]. It is possible that melittin may also act as a cationic protein to trigger bilayer-to-non-bilayer transitions, leading to the formation of transient compartments in the intra-crista space. However, this hypothesis requires experimental substantiation.

The movement of protons along the inner mitochondrial membrane (IMM) surface within the intra-cristae space and through the F_o_ subunit is critical for establishing a pH gradient between F_o_ and F_1_ [52]. This gradient is required to release the ATP synthase rotor from its inhibited state, in which it is held by one CL molecule ionic bound to conserved Lys7 on the intra-cristae side and another CL bound to conserved Lys43 on the matrix side [65,66]. Proton flow neutralizes the phosphate groups of these CL molecules, thereby unlocking the rotor and restarting ATP synthesis.

Although it has been proposed that protons passing through the F_o_ subunit do not disperse into the surrounding aqueous environment [52], the destination of these protons—and thus the maintenance of the pH gradient—has remained unclear. In our recent work [26,30,63], we have proposed that protons traversing the F_o_ subunit not only neutralize CL phosphate groups to release the rotor, but also induce a conical conformation in CL molecules, prompting their transformation into small, inverted micelles within the hydrophobic core of the IMM. This process encapsulates protons within the hydrophilic interior of the micelles as they emerge from the F_o_ subunit, thereby preserving the pH gradient between F_o_ and F_1_ and sustaining proton flow through the F_o_ subunit. In this way, inverted micelles act as nanocarriers, shuttling protons across the low-dielectric alkyl chain region of the IMM toward the proton pumps of respiratory complexes [30].

**Figure 12:**
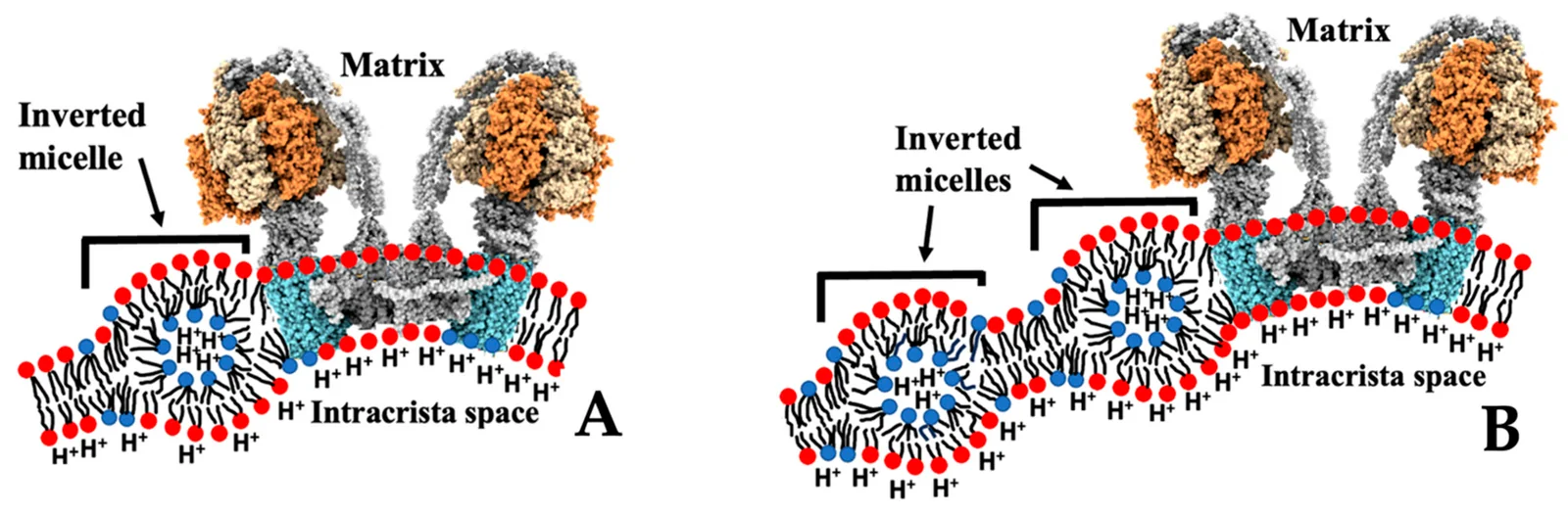
Formation of inverted micelles acting as nanocarriers for proton transfer from the F_o_ subunit of ATP synthase to respiratory complexes in the IMM. As protons pass through the F_o_ unit of ATP synthase, they increase the conical curvature of cardiolipin molecules, triggering the formation of inverted micelles that carry H^+^ ions (**A**) across the hydrophobic core of the IMM toward the nearest proton pump of a respiratory complex. This micelle formation is coupled with the extraction of CL from conserved Lys7 and Lys43 residues on the rotor, facilitating rotor spinning and ATP production. Newly generated inverted micelles, in turn, push preexisting ones (**B**) further toward the proton pump. The structural reconstitution of ATP synthase and the phospholipid head color coding are as described in Figure 10. Schematics (**A**,**B**) are adapted from [30].

Following proton transfer, the deprotonated CL molecules move along the IMM surface on the intra-crista side and return to the F_o_ subunit [67], where they re-bind to the conserved Lys7 and Lys43 residues on the rotor. However, shortly thereafter, a new surge of protons moving through the F_o_ subunit again neutralizes the phosphate groups of CL at these residues, unlocking the rotor and triggering the formation of another inverted micelle that ferries protons to the respiratory complex proton pumps (Figure 12).

Our AutoDock studies have shown that Arg22 of native melittin strongly targets the CL polar head in five out of six binding sites on the molecular surface of melittin that involve polar-ionic and hydrogen bonds (Table 1). In three of these sites, Lys23 accompanies Arg22 in targeting the CL polar head. Notably, Arg22 predominantly targets the phosphate groups of CL, while Lys23 targets the ester carbonyl (-C=O) group.

It is highly compelling to hypothesize that native melittin would target CL ionically bound to conserved Lys7 and Lys43 residues of the ATP synthase rotor to overtake CL from these rotor lysines. Arg22 of melittin most likely acts in synergy with Lys23. This synergy is plausible given their complementary chemical properties: Lys23 provides a highly concentrated, localized positive charge for a strong primary polar-ionic grip, while the guanidinium group of Arg22 offers a superior binding advantage through its delocalized charge and bidentate hydrogen-bonding capacity, enabling a more versatile and desolvated secondary grip. Together, these features would allow melittin to “overtake” CL from the rotor’s Lys7 and Lys43 residues and promote ATP synthesis. Additionally, the extensive hydrophobic areas on the molecular surface of melittin bind tightly to the alkyl chains of CL, as shown by our AutoDock study, which would further facilitate the detachment of CL from the rotor.

By targeting Lys7 and Lys43 of the rotor, native melittin not only detaches CL from the rotor but also induces a conical shape in CL, leading to the formation of inverted micelles. These inverted micelles entrap protons within their hydrophilic core, where amphiphilic melittin would be ideally positioned at the interface between the hydrophilic and hydrophobic regions of the micelle. This assumption is supported by our proton adsorption results in PC + CL membranes treated with melittin, where proton adsorption increased exponentially at CL-to-melittin molar ratios of 80 and higher.

Melittin itself does not directly contribute to proton adsorption on the membrane surface; rather, it triggers the formation of non-bilayer structures that adsorb protons by entrapping them within the hydrophilic core of inverted micelles. Thus, melittin enhances mitochondrial ATP synthesis by unlocking the ATP synthase rotor and by removing protons from the F_o_ subunit, which helps maintain the pH gradient between F_o_ and F_1_ and facilitates proton movement through the F_o_ subunit. The inverted micelles may act as nanocarriers, shuttling protons through the hydrophobic core of the inner mitochondrial membrane to the proton pump of a respiratory complex.

Notably, modified melittin—which lacks Arg22—does not trigger the formation of non-bilayer structures, does not enhance mitochondrial ATP production, and, according to AutoDock predictions, does not involve Lys23 in interaction with the CL polar head. These findings support our hypothesis that the Arg22 residue of melittin acts in tandem with Lys23 to unlock the ATP synthase rotor.

It is important to emphasize that the decreased activity in our experimental settings observed with the Arg22 → Gly22 modified melittin does not imply that Arg22 alone is responsible for the entire molecular activity of melittin. Rather, our data indicate that Arg22 is a key component of a cooperative interaction network involving additional residues such as Lys23 and, to a lesser extent, Trp19, Lys21, and Gln26. The substitution likely affects the overall protein–lipid interface, upsetting the binding geometry of melittin with the CL-containing membrane. Further mutational, quantitative binding and structural studies are needed to fully resolve the contributions of individual residues and the role of the entire sequence.

Our findings align well with the structure and pharmacological activities of the novel potential pharmaceutical Szeto–Schiller (SS) tetrapeptides, which are being designed to mitigate or even reverse the decline in mitochondrial bioenergetics associated with aging and disease [68]. These amphipathic cationic tetrapeptides contain alternating Arg and Lys residues with hydrophobic aromatic amino acids. They are designed to penetrate cellular and organellar membranes and target mitochondrial CL. To increase resistance to aminopeptidase degradation, the Arg residue is typically present as the D-enantiomer rather than the L-enantiomer.

SS tetrapeptides have been shown to rejuvenate mitochondrial morphology and bioenergetics in animal models; however, their precise mechanism of pharmacological action has not yet been proposed [62,68]. We have synthesized the SS-31 tetrapeptide (Arg–MT–Lys–Phe–NH_2_, where MT is 2′,6′-dimethyltyrosine) with both Arg and MT as L-enantiomers, whereas in the original SS-31 peptide, both are D-enantiomers. Our unpublished data show that 0.5 × 10^−7^ M of the L-enantiomer SS-31 triggers the formation of non-bilayer structures in CL-containing membranes and enhances mitochondrial ATP production.

Like melittin, the SS-31 tetrapeptide has an amphipathic structure with Arg and Lys residues positioned in close proximity. It is plausible that the therapeutic activity of SS-31 observed in animals [62,68] is based on a mechanism similar to the one we propose here for native melittin: unlocking the ATP synthase rotor to promote ATP production by targeting CL molecules bound to Lys7 and Lys43 of the rotor and removing detached CL and protons from the F_o_ subunit via inverted micelles.

Overall, the comparison with Szeto-Schiller (SS) peptides is intended to contextualize the mechanistic importance of Arg–Lys cooperativity in cardiolipin binding. Like SS-31, melittin presents an amphipathic architecture with Arg and Lys residues in close proximity, which our AutoDock simulations identify as a key to CL targeting. However, this analogy is limited to these specific structural and mechanistic features and does not imply that melittin shares the pharmacological profile, safety, or therapeutic utility of SS-31. Melittin is not proposed here as a therapeutic agent. Rather, our findings may inform the design of synthetic CL-targeting molecules that mimic specific elements of the melittin–CL interaction, such as the cooperative action of Arg and Lys residues, while any translational application would require extensive independent validation.

## 4. Conclusions

In this study, we have shown that Arg22 of bee venom melittin, in synergy with Lys23, plays an important role in targeting the charged and polar groups of cardiolipin (CL) to trigger the formation of inverted micelles in CL-containing membranes. These inverted micelles adsorb protons by entrapping them within their hydrophilic core, which is formed in the hydrophobic center of the membrane.

Based on the array of computational and experimental data obtained in this work, together with published findings, we propose that Arg22, in tandem with Lys23, targets and neutralizes CL bound to conserved Lys7 and Lys43 of the ATP synthase rotor, thereby detaching CL from the rotor. These events promote a conical shape of CL, which triggers the formation of inverted micelles that entrap protons from the F_o_ subunit and transfer them to the proton pump of a respiratory complex.

The findings of this study have significant potential to advance our understanding of the mechanisms underlying mitochondrial energetics. The results also pave the way for new applications of natural toxins in the design of novel pharmaceuticals aimed at treating medical conditions associated with the decline in mitochondrial energy production during aging and/or disease.

## 5. Materials and Methods

### 5.1. Materials

All cloning materials, including the expression vector pGEX-6P-1, the TA cloning vector pMD19-T, restriction enzymes, T4 DNA ligase, DNA marker, protein molecular weight marker, Plasmid Mini kit I, and the gel extraction kit, were purchased from Shengwufeng Biotech (BioWind), China. *E. coli* DH5α competent cells were used for cloning and sequence verification, and *E. coli* BL21(DE3) cells (Invitrogen, Carlsbad, CA, USA) were used as the bacterial expression host. Ampicillin was obtained from Anhui Haiyang Pharmaceutical Co., Ltd. (Bengbu City, China) and IPTG was obtained from Beijing Solarbio Science & Technology Co., Ltd. (Beijing, China). Primers and the native and modified melittin nucleotide sequences were synthesized by Shengwufeng Biotech, Shanghai, China. The tagged proteins were purified with Glutathione Sepharose High-Performance medium (GE Healthcare, Piscataway, NJ, USA). The tagged proteins were digested with Prescission protease (PPase) (APExBIO Technology LLC—based in Houston, TX, USA). The supernatants from the digestion mixture were fractionated on a Jupiter C_5_ column (4.6 × 150 mm, 30 nm pore size, 5 μm particle size; Phenomenex, Torrance, CA, USA). Samples were centrifuged on Sorvall ST 8 compact centrifuge (Thermo Scientific, Waltham, MA, USA). Molecular weight markers for SDS-PAGE on a 14% gel were from Takara (Dalian, China). Molecular weight markers for SDS-PAGE on an 18% gel were from Precision Plus Protein^TM^ Dual Xtra Standards (Bio-Rad, Carlsbad, CA, USA). SDS-PAGE runs were completed on The Mini-PROTEAN Tetra Vertical Electrophoresis Cell using the Mini-PROTEAN TGX precast gels of 14% or 18% density. Circular dichroism spectra were recorded on a Jasco J-810 spectropolarimeter (JASCO, Inc., Easton, MD, USA). Proteins were lyophilized on vacuum freeze-drying lyophilizing machine YTLG-10 (Shanghai Yetuo Co., Ltd., Shanghai, China). Lipids were sonicated on Ultrasonic Dispenser Yt-JY96-IIN (Shanghai Yetuo technology Co., Ltd., Shanghai, China); the pH Meter PHS-3C was obtained from Shanghai INESA Scientific Instruments Co., Ltd., Shanghai, China. Polymorphism of PC in PC + CL membrane was studied and phospholipid concentration in mitochondrial samples was determined by using NMR Varian XL-200 spectrometer (Varian Instruments, Palo Alto, CA, USA). Specifications for recording ^1^H-NMR and ^31^P-NMR spectra were previously described [31]. Bee venom melittin (98% purity) was obtained from GenScript Co., Ltd. (Nanjing, China). Bovine liver cardiolipin (CL) and egg yolk L-α-phosphatidylcholine (PC) were obtained from Merck KGaA (Darmstadt, Germany).

### 5.2. Computational Docking of Native and Modified Melittins with Cardiolipin

To identify predicted docking sites for CL on the molecular surface of melittin and to determine the tentative charged and polar groups of amino acid residues involved in intermolecular bonds with the charged and polar moieties of CL, we docked melittin with the polar head group of CL using AutoDock Vina version 4.2 [40]. Digital coordinates for *Apis mellifera* melittin monomer—referred to in this article as native melittin—were extracted from the digital melittin dimer (PDB entry 2MLT). Coordinates for CL were obtained from the bovine heart oxidoreductase crystal structure bound to CL (PDB entry 1V54). The alkyl chains of CL were removed using Avogadro editor 2.0.0 [69]. Modified melittin, in which glycine substitutes for arginine at position 22, was generated by deleting the Arg22 side chain using PyMOL^TM^ 2.5.5.

The overall charges of the digital molecules were verified and energy-minimized using AutoDock. All covalent bonds in the CL polar head were set as rotatable, while all bonds in native and modified melittin were kept non-rotatable. A grid box with the following dimensions—center x = 28.79, center y = −1.967, center z = 16.698; size x = 34.5 Å, size y = 25.5 Å, size z = 16.5 Å—encompassed the entire molecular surface of melittin.

For each AutoDock run, the program generated the nine lowest-binding-enthalpy melittin–CL docked conformations, which we analyzed for binding site locations and types of intermolecular bonds using Python Molecular Viewer version 1.5.7. The exhaustiveness setting was set up as 16, which produced consistent results for docking of CL with both native and modified melittin completed in triplicate runs. No deviation was observed among the docked conformations and intermolecular bondings within the triplicate runs.

### 5.3. Construction of Expression Vectors

The melittin coding sequences (native melittin and r-modified melittin (Arg22 → Gly22) were codon-optimized for *E. coli* and synthesized by Shengwufeng Biotech, Shanghai, China. The native and r-modified inserts were PCR-amplified with Taq polymerase using a shared forward primer that introduced a BamHI site and a construct-specific reverse primer that introduced a TAA stop codon followed by an EcoRI site (Table 5). The Arg22 → Gly22 substitution was introduced through the reverse primer. The PCR products were first cloned into pMD19-T (TaKaRa) and verified by Sanger sequencing. The verified inserts were then excised with BamHI and EcoRI (Shengwufeng Biotech, Shanghai, China), ligated with T4 DNA ligase Shengwufeng Biotech, Shanghai, China) into pGEX-6P-1 (Shengwufeng Biotech, Shanghai, China) digested with the same enzymes, and transformed into *E. coli* DH5α. The final constructs were confirmed by Sanger sequencing using the pGEX 5′ and 3′ primers.

The inserts were designed to be in frame with the upstream glutathione S-transferase (GST) tag and the HRV 3C (PreScission) protease cleavage site (LEVLFQ↓GP), and the TAA stop codon, placed immediately before the EcoRI site, terminates translation at the C-terminus. After PreScission cleavage, each recombinant peptide was therefore expected to carry the vector-derived N-terminal residues GPLGS (encoded by the cleavage site and the BamHI junction), followed by the r-native or r-modified melittin sequence, with no additional C-terminal vector residues.

### 5.4. Expression of Recombinant Native GST-Melittin and Modified GST-Melittin

The pGEX-6P-1 plasmids encoding native GST–melittin and modified (Arg22 → Gly22) GST–melittin were propagated in *E. coli* DH5α, and their inserts were verified by Sanger sequencing using pGEX 5′ and 3′ sequencing primers (binding sites flanking the MCS of pGEX-6P-1). Each verified plasmid was then transformed into the expression host *E. coli* BL21(DE3) by heat shock, and transformants were selected on LB agar containing 100 µg/mL of ampicillin. For expression, a single colony of each strain was inoculated into 250 mL of LB broth (Solarbio, Beijing, China) containing 100 µg/mL ampicillin in a 500 mL flask and grown at 37 °C with shaking at 180 rpm until the OD600 reached 0.6. Expression was induced with 0.1 mM of IPTG (Solarbio, Beijing, China) and the temperature was lowered to 25 °C for approximately 18 h with shaking at 180 rpm. Cells were harvested by centrifugation at 8000× *g* for 10 min at 4 °C and resuspended in 5 mL of buffer A1 (140 mM of NaCl, 2.7 mM of KCl, 10 mM of Na_2_HPO_4_, 1.8 mM of KH_2_PO_4_, pH 7.3). The cells were lysed on ice by ultrasonication using an intelligent ultrasonic processor (YT-JY96-IIN, 150 W, Shanghai YETO Instrument & Meter Co., Ltd., Shanghai, China). The lysate was clarified by centrifugation at 10,000× *g* for 45 min at 4 °C, and the supernatant containing the soluble GST–melittin fusion protein was collected for purification.

### 5.5. Purification of Fusion Proteins: Native GST-Melittin and Modified GST-Melittin

The supernatants from the lysates were filtered through a 0.22 μm microporous membrane and loaded onto separate columns containing Glutathione Sepharose High-Performance medium (GE Healthcare, Piscataway, NJ, USA) equilibrated with buffer A1. The columns were washed with approximately 15 mL of buffer A1 at a flow rate of 1 mL/min to remove untagged proteins. The tagged proteins—native GST–melittin or modified GST–melittin—were then eluted with buffer B1 (50 mM of Tris-HCl, 10 mM of reduced glutathione, pH 8.0). The eluted proteins were collected, dialyzed, and lyophilized prior to analysis by SDS-PAGE on a 14% gel.

### 5.6. Purification of Recombinant Native and Modified Melittins

The two samples of mixed proteins eluted from the separate columns described above were each treated with Prescission protease (PPase) in buffer A2 (50 mM of Tris-HCl, 150 mM of NaCl, 1 mM of EDTA, 1 mM of dithiothreitol, pH 7.4) for 10 h at 4 °C. Following digestion, the PPase in each sample was pelleted by centrifugation at 500× *g* for 5 min, and the supernatants were collected, dialyzed, and lyophilized prior to further fractionation by reverse-phase high-performance liquid chromatography on a Jupiter C_5_ column (4.6 × 150 mm, 30 nm pore size, 5 μm particle size; Phenomenex).

Chromatographic separation was performed using a linear gradient of acetonitrile in 0.1% TFA at a flow rate of 1 mL/min, as follows: 0–20% acetonitrile over the first 10 min, 20–60% over the next 60 min, and 60–80% over the final 10 min. Eluate absorbance was monitored at 280 nm. Recombinant melittins—native or modified—eluted in the final fractions of each fractionation were collected, dialyzed, and lyophilized prior to analysis by SDS-PAGE on an 18% gel and by circular dichroism.

### 5.7. Circular Dichroism Analysis of r-Native and r-Modified Melittin

Circular dichroism (CD) spectra of recombinant native melittin, recombinant modified melittin, and commercial bee venom melittin were recorded at a protein concentration of 10 µM in phosphate-buffered saline (1.47 mM of KH_2_PO_4_, 4.29 mM of Na_2_HPO_4_, 137 mM of NaCl, 2.68 mM of KCl, pH 7.4) using a Jasco J-810 spectropolarimeter (JASCO, Inc., Easton, MD, USA). Wavelength scans were collected over the range of 170–300 nm in phosphate-buffered saline. The following parameters were used for each set of 30 scans: response time, 1 s; sensitivity, 100 mdeg; and scan speed, 50 nm/min.

### 5.8. Proton Adsorption on the Surface of PC + CL Membranes Modulated by r-Native, Commercial and r-Modified Melittins

Liposomes composed of PC + 20 mol%CL, at a total phospholipid concentration of 6 × 10^−8^ M, were prepared by sonication in saline (154 mM of NaCl) at pH 6.88. The pH values in bulk water were measured with a pH Meter PHS-3C (Shanghai INESA Scientific Instruments Co., Ltd., Shanghai, China). Phospholipids were first dissolved in a 1:1 (*v*/*v*) mixture of chloroform and methanol. For liposomes treated with melittin, the required concentration of melittin was added to the phospholipid solution in the chloroform/methanol mixture. The organic solvents were then evaporated under vacuum at 10 Pa for 2 h at 25 °C.

The resulting phospholipid film was hydrated with saline at pH 6.88 and sonicated using an ultrasonic disperser on ice at a frequency of 22 kHz for 15 min to produce unilamellar liposomes. Prior to pH measurements, all samples—both saline and liposome suspensions—were incubated at 25 °C for 30 min to standardize conditions for temperature and to allow for protons in bulk water and those adsorbed on the membrane surface to reach equilibrium.

Proton concentrations were calculated using the conversion [H^+^] = 10^−pH^. The concentration of protons adsorbed by the liposomal membrane was determined by subtracting the [H^+^] in the liposome-containing saline solution from that in pure saline solution. All measurements were performed in triplicate, and the standard deviation between measurements never exceeded ±5%.

### 5.9. Effects of r-Native, Commercial, and r-Modified Melittins on Mitochondrial ATP-Synthase Activity

To isolate bovine heart mitochondria, we followed sequential centrifugation steps [70,71] with minor modifications. Briefly, at the final step the mitochondrial pellet was washed by re-suspension with the ice-cold washing medium containing 1 mM of EDTA, 0.3 M of mannitol, 10 mM of MOPS, and 0.1% (*w*/*v*) BSA at pH 7.4. Mitochondrial protein concentration in mg/mL was determined using the Bradford method [72]. To determine the absolute phospholipid content in each mitochondrial sample, we compared the integrated ^31^P-NMR signal intensities of the samples against those of multilamellar liposomes with known phospholipid concentrations as described previously [31,73]. Mitochondrial ATP synthase activity, which is the ATP levels in mitochondria, expressed as µmol ATP synthesized per mg of mitochondrial proteins, was measured according to the protocol previously described [74]. The phospholipid concentration in each mitochondrial sample was estimated to be 3.2 × 10^−2^ M, which corresponds to approximately 0.64 × 10^−2^ M CL. Mitochondrial ATP synthase activity was measured at 37 °C in the presence of 1.5 mM succinate. Measurements were performed in a control sample (no melittin) and in samples treated with r-native melittin, commercial melittin, or r-modified melittin at concentrations of 3.20 × 10^−5^ M, 6.40 × 10^−5^ M, 1.28 × 10^−4^ M, 2.56 × 10^−4^ M, 5.33 × 10^−4^ M, or 1.07 × 10^−3^ M. These concentrations correspond to CL-to-melittin molar ratios of 200, 100, 50, 25, 12, and 6, respectively. Samples were incubated for 10 min at 37 °C prior to ATP level measurements. The ATP synthase activity of each mitochondrial sample was calculated from the triplicate measurements. To minimize technical variability, all mitochondrial samples were prepared from a single stock of isolated bovine heart mitochondria. The inter-sample variation was less than ±4%, and inter-experimental variability remained below ±5%.

### 5.10. ^1^NMR Studies of the Abilities of r-Native Melittin, Bee Venom Melittin and r-Modified Melittin to Induce Non-Bilayer Structures in PC + 20 mol%CL Liposomes

Unilamellar PC + 20 mol% CL liposomes for ^1^H-NMR studies were prepared by drying phospholipids from a chloroform/methanol mixture (1:1 *v*/*v*) under vacuum at 10 Pa for 2 h at 25 °C to form a lipid film. The film was then hydrated in a ^2^H_2_O buffer containing 10 mM of Tris-HCl, pH 6.88, and 0.5 mM of EDTA. The lipid suspension was sonicated for 15 min using an ultrasonic disperser on ice at a frequency of 22 kHz. Unilamellar liposomes were centrifuged at 200× *g* for 60 min to remove heavy phospholipid aggregates and then incubated under a helium atmosphere for 15 h at 10 °C. The total phospholipid concentration in the unilamellar liposome suspension was 1.5 × 10^−2^ M, and the concentration of melittin (recombinant native, commercial bee venom, or recombinant modified) was either 1.2 × 10^−4^ M or 3.0 × 10^−4^ M corresponding to CL-to-melittin molar ratios of either 25 or 100, respectively.

All ^1^H-NMR spectra of liposomes were recorded in the presence of K_3_[Fe(CN)_6_] (10 µL of saturated K_3_[Fe(CN)_6_] solution per 1 mL of liposome suspension). Liposome samples were first incubated for 15 min at 25 °C, followed by the addition of K_3_[Fe(CN)_6_] and a further 5-min incubation before the addition of melittin. ^1^H-NMR spectra were acquired 5 min after melittin addition. Spectra were recorded at 25 °C at an operating frequency of 200 MHz. The 90° pulse width was 8.7 µs, the relaxation delay was 50 µs, and the acquisition time for the free induction signal was 1 s.

Samples for each experimental condition were prepared three times, and ^1^H-NMR spectra for each sample were acquired in triplicate. Triplicate measurements for each condition yielded nearly identical results.

### 5.11. Statistics

The numerical results are expressed as mean ± standard deviation from three independent experiments. Data were analyzed using Student’s *t*-test (two-tailed) for single comparisons, while multiple group comparisons were performed using one-way ANOVA with Tukey’s post hoc test. A *p*-value of less than 0.05 was considered statistically significant.

## Figures and Tables

**Figure 3:**
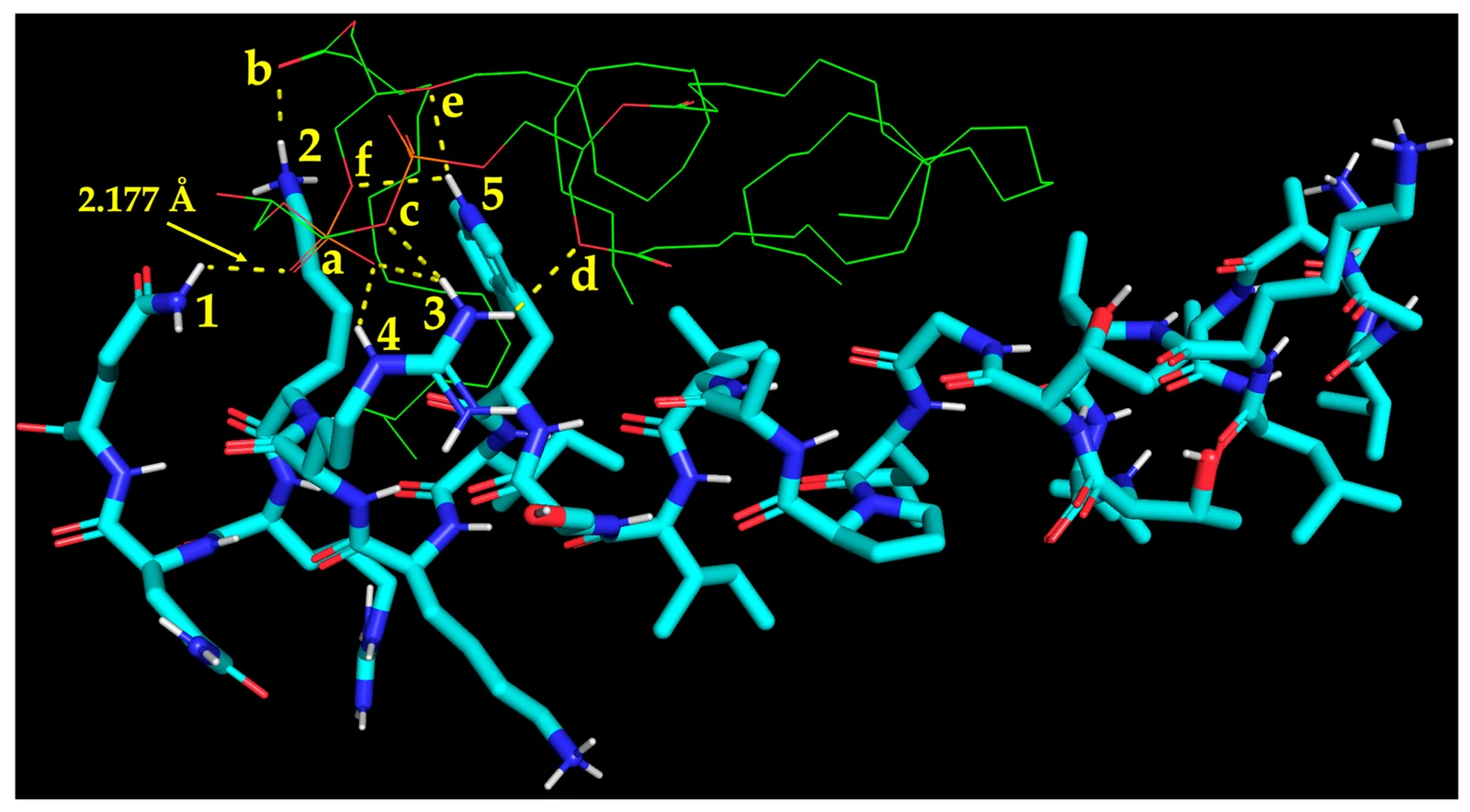
Binding site #3 for CL on the molecular surface of native melittin, as predicted by AutoDock Vina simulation. Melittin is rendered in stick representation, with carbon in emerald, oxygen in red, hydrogen in white, and nitrogen in blue. CL is rendered in line representation, with carbon in green, phosphorus in orange, and oxygen in red; hydrogen atoms are omitted for visual clarity. Intermolecular bonds are depicted as yellow dashed lines. The polar and charged moieties of melittin involved in intermolecular bonds are labeled with numbers, and those of CL are labeled with letters, following the same labeling scheme used in Table 1. The lengths of the intermolecular bonds ranged from 2.177 to 2.384 Å.

**Table 2:** Binding site affinity energies, polar and charged moieties of modified melittin and the CL polar head, and types of intermolecular bonds predicted by AutoDock Vina simulation. Notations for the polar and charged groups in the CL polar head are provided in Figure 2.

Number of Binding Site and Binding Affinity	Polar & Charged Groups in Modified Melittin	Polar & Charged Groups in CL	Bond Type
#1 −10.45 kJ/mol	Ser18(COH^δ+^) Trp19(CNH^δ+^C)	B-CO^δ−^P II-C=O^δ−^	Hydrogen Hydrogen
#2 −10.45 kJ/mol	Trp19(CNH^δ+^C)	1-CO^δ−^C	Hydrogen
#3 −10.45 kJ/mol	Gln26(O=CNH_2_^δ+^) Ser18(COH^δ+^)	A-PO_4_^−^ B-PO_4_^−^	Polar-ionic Hydrogen
#4 −10.45 kJ/mol	Lys21(CN^+^H_3_)	A-CO^δ−^P	Polar-ionic
#5 −10.03 kJ/mol	Lys21(CN^+^H_3_)	1-CO^δ−^C	Polar-ionic
#6 −10.03 kJ/mol			Non-polar
#7 −10.03 kJ/mol	Lys21(CN^+^H_3_)	A-PO_4_^−^	Ionic
#8 −10.03 kJ/mol			Non-polar
#9 −10.03 kJ/mol			Non-Polar

**Table 3:** Adsorption of H^+^ onto the surface of PC liposomes containing 20 mol% CL and treated with increasing concentrations of r-native melittin. Total lipid concentration was 6 × 10^−8^ M and CL concentration was 1.2 × 10^−8^ M. All pH measurements were taken at 25 °C. The concentration of H^+^ adsorbed by the liposomes was determined by subtracting the [H^+^] in the liposome-containing saline solution from that in pure saline solution. The molar ratio of H^+^ adsorbed by liposomes per 1.2 × 10^−8^ M of CL is provided in the first sub-column of the last column. The first row in the first sub-column of the last column corresponds to pure liposomes (no melittin), while the subsequent rows represent liposomes with varying molar ratios of CL to r-native melittin (MLT).

pH in Saline	[H^+^] in Saline, (M)	pH in Saline with Liposomes	[H^+^] in Saline with Liposomes, (M)	[H^+^] Adsorbed by Liposomes in Saline, (M)	Molar Ratio of Adsorbed Protons (H^+^) per CL *, Molar Ratio of CL to MLT ^†^, and MLT ^†^ Concentrations		
6.880 ± 0.001	1.3180 × 10^−7^	6.896 ± 0.001	1.2700 × 10^−7^	0.0480 × 10^−7^	0.4000	CL/MLT ^†^	MLT ^†^ (M)
6.880 ± 0.001	1.3180 × 10^−7^	6.896 ± 0.001	1.2700 × 10^−7^	0.0480 × 10^−7^	0.4000	200	6.00 × 10^−11^
6.880 ± 0.001	1.3180 × 10^−7^	6.896 ± 0.004	1.2700 × 10^−7^	0.0480 × 10^−7^	0.4000	170	7.05 × 10^−11^
6.880 ± 0.001	1.3180 × 10^−7^	6.896 ± 0.003	1.2700 × 10^−7^	0.0480 × 10^−7^	0.4000	140	8.57 × 10^−11^
6.880 ± 0.001	1.3180 × 10^−7^	6.896 ± 0.002	1.2700 × 10^−7^	0.0480 × 10^−7^	0.4000	110	1.09 × 10^−10^
6.880 ± 0.001	1.3180 × 10^−7^	6.928 ± 0.008	1.1796 × 10^−7^	0.1384 × 10^−7^	1.1320	80	1.50 × 10^−10^
6.880 ± 0.001	1.3180 × 10^−7^	6.992 ± 0.014	1.0133 × 10^−7^	0.3047 × 10^−7^	2.5390	50	2.40 × 10^−10^
6.880 ± 0.001	1.3180 × 10^−7^	8.247 ± 0.191	0.0566 × 10^−7^	1.2614 × 10^−7^	10.520	20	6.00 × 10^−10^
6.880 ± 0.001	1.3180 × 10^−7^	8.439 ± 0.230	0.0364 × 10^−7^	1.2816 × 10^−7^	10.680	10	1.20 × 10^−9^
6.880 ± 0.001	1.3180 × 10^−7^	8.476 ± 0.223	0.0334 × 10^−7^	1.2846 × 10^−7^	10.705	5	2.40 × 10^−9^

* Molar ratio of adsorbed protons to CL was determined by dividing [H^+^] adsorbed by liposomes by CL concentration (1.2 × 10^−8^ M). ^†^ R-native melittin. Each pH value represents the mean of triplicate measurements ± SD. ANOVA with Tukey’s test was used to compare means between groups treated with different melittins and Student’s *t*-test was used to compare means for the groups treated with the same type of melittin. A *p*-value < 0.05 was considered statistically significant.

**Table 4:** Adsorption of H^+^ onto the surface of PC liposomes containing 20 mol% CL and treated with increasing concentrations of r-modified melittin. Total lipid concentration was 6 × 10^−8^ M and CL concentration was 1.2 × 10^−8^ M. All pH measurements were taken at 25 °C. The concentration of H^+^ adsorbed by the liposomes was determined by subtracting the [H^+^] in the liposome-containing saline solution from that in pure saline solution. The molar ratio of H^+^ adsorbed by liposomes per 1.2 × 10^−8^ M of CL is provided in the first sub-column of the last column. The first row in the first sub-column of the last column (no colored) corresponds to pure liposomes, while the subsequent rows represent liposomes with varying molar ratios of CL to r-modified melittin (MLT).

pH in Saline	[H^+^] in Saline, (M)	pH in Saline with Liposomes	[H^+^] in Saline with Liposomes, (M)	[H^+^] Adsorbed by Liposomes in Saline, (M)	Molar Ratio of Adsorbed Protons (H^+^) per CL *, Molar Ratio of CL to MLT ^†^, and MLT ^†^ Concentrations		
6.880 ± 0.001	1.3180 × 10^−7^	6.896 ± 0.003	1.2700 × 10^−7^	0.0480 × 10^−7^	0.400	CL/MLT ^†^	MLT ^†^ (M)
6.880 ± 0.001	1.3180 × 10^−7^	6.896 ± 0.003	1.2700 × 10^−7^	0.0480 × 10^−7^	0.400	200	6.00 × 10^−11^
6.880 ± 0.001	1.3180 × 10^−7^	6.896 ± 0.005	1.2700 × 10^−7^	0.0480 × 10^−7^	0.400	170	7.05 × 10^−11^
6.880 ± 0.001	1.3180 × 10^−7^	6.896 ± 0.005	1.2700 × 10^−7^	0.0480 × 10^−7^	0.400	140	8.57 × 10^−11^
6.880 ± 0.001	1.3180 × 10^−7^	6.896 ± 0.005	1.2700 × 10^−7^	0.0480 × 10^−7^	0.400	110	1.09 × 10^−10^
6.880 ± 0.001	1.3180 × 10^−7^	6.897 ± 0.003	1.2692 × 10^−7^	0.0488 × 10^−7^	0.408	80	1.50 × 10^−10^
6.880 ± 0.001	1.3180 × 10^−7^	6.897 ± 0.006	1.2689 × 10^−7^	0.0491 × 10^−7^	0.408	50	2.40 × 10^−10^
6.880 ± 0.001	1.3180 × 10^−7^	6.898 ± 0.004	1.2682 × 10^−8^	0.0498 × 10^−7^	0.415	20	6.00 × 10^−10^
6.880 ± 0.001	1.3180 × 10^−7^	6.897 ± 0.005	1.2676 × 10^−8^	0.0504 × 10^−7^	0.420	10	1.20 × 10^−9^
6.880 ± 0.001	1.3180 × 10^−7^	6.898 ± 0.004	1.2652 × 10^−8^	0.0528 × 10^−7^	0.440	5	2.40 × 10^−9^

* Molar ratio of adsorbed protons to CL was determined by dividing [H^+^] adsorbed by liposomes by CL concentration (1.2 × 10^−8^ M). ^†^ R-modified melittin. Each pH value represents the mean of triplicate measurements ± SD. ANOVA with Tukey’s test was used to compare means between groups treated with different melittins and Student’s *t*-test was used to compare means for the group treated with the same type of melittin. A *p*-value < 0.05 was considered statistically significant.

**Table 5:** Primers used for cloning melittin inserts.

Construct	Forward Primer (5′ → 3′)	Reverse Primer (5′ → 3′)
r-native melittin	GCGGATCCGGTATCGGTGCGGT TCTGAAAGTTCTG	GCGAATTCTTACTGCTGACGTTTA CGTTTGATCCAAGAGAT
r-(Arg22 → Gly22) melittin *	GCGGATCCGGTATCGGTGCGGT TCTGAAAGTTCTG	GCGAATTCTTACTGCTGACGTTTA CCTTTGATCCAAGAGAT

* r-modified melittin.

## Data Availability

The original contributions presented in this study are included in the article/Supplementary Materials. Further inquiries can be directed to the corresponding author.

## Supplementary Materials

The following supporting information can be downloaded at: https://www.mdpi.com/article/10.3390/toxins18100432/s1, Figure S1. Three-dimensional schematic representation of the amino acid sequences of native melittin (A) and modified melittin with Arg22 substituted by Gly22 (B), shown in line representation. Carbon atoms are shown in green, oxygen in red, hydrogen in white, and nitrogen in blue. Figure S2. Binding site #1 for CL on the molecular surface of modified melittin, as predicted by AutoDock Vina simulation. Modified melittin is shown in line representation, with carbon in green, oxygen in red, hydrogen in white, and nitrogen in blue. CL is rendered in stick representation, with carbon in emerald, phosphorus in orange, and oxygen in red; hydrogen atoms are omitted for visual clarity. Intermolecular bonds are depicted as yellow dashed lines. Figure S3. SDS-polyacrylamide (14%) gel electrophoresis of eluates from a glutathione Sepharose affinity column eluted with buffer B. Lane 1: eluate containing GST-native melittin; Lane 2: eluate containing GST-modified melittin; Lane 3: molecular weight markers (Takara, Dalian, China). Additional lanes show unrelated supernatant samples from lysed cells filtered through a 0.22 μm microporous membrane; these samples are not individually labeled. Figure S4. SDS-polyacrylamide (18%) gel electrophoresis of r-modified melittin (lane 2), r-native melittin (lane 3) and commercial melittin (98%purity) (lane 4). Lane 1: molecular weight markers from Presicion Plus ProteinTM Dual Xtra Standards (Bio-Rad, Carlsbad, CA, USA). Additional lanes, which are not individually labeled, show unrelated samples. Table S1. Adsorption of H^+^ onto the surface of PC liposomes containing 20 mol% CL, at a total lipid concentration of 6 × 10^−8^ M and concentration of CL 1.2 × 10^−8^ M. All pH measurements were taken at 25 °C. The concentration of H^+^ adsorbed by the liposomes was determined by subtracting the [H^+^] in the liposome-containing saline solution from that in pure saline solution. The molar ratio of H^+^ adsorbed by liposomes per 1.2 × 10^−8^ M of CL is provided in the first sub-column of the last column (in yellow). The first row in the first sub-column of the last column (no color) corresponds to pure liposomes (no melittin), while the subsequent rows represent liposomes with varying molar ratios of CL to bee venom melittin (MLT).

## Abbreviations

The following abbreviations are used in this manuscript:

MLT	Melittin
CL	Cardiolipin
PC	Phosphatidylcholine
PE	Phosphatidylethanolamine
SM	Sphingomyelin
PS	Phosphatidylserine
PI	Phosphatidylinositol
Arg22	Arginine 22
Gly22	Glycine 22
Lys43	Lysine 43
Gln26	Glutamine 26
Trp19	Tryptophane 19
Ser18	Serine 18
ATP	Adenosine triphosphate
ADP	Adenosine diphosphate
IMM	Inner mitochondrial membrane
GST	Glutathione-S-transferase
IPTG	Isopropyl β-D-thiogalactoside
SDS-PAGE	Sodium dodecyl phosphate polyacrylamide gel electrophoresis
Ppase	Prescission protease
CD	Circular dichroism
NMR	Nuclear magnetic resonance
SS	Szeto–Schiller tetrapeptide
DNA	Deoxynucleic acid
HRV	2.3 Human Rhinovirus
PCR	Polymerase chain reaction
OD	Optical density
EDTA	Ethylenediaminetetraacetic acid
MOPS	3-(N-morpholino)propanesulfonic acid
