# Supplementary material for: Cardiolipin-Specific Targeting of Melittin via Arg22 in Lipid Membranes: A Natural Toxin-Based Strategy for Treating Mitochondrial Dysfunction in Aging and Disease
Source: Toxins (Basel). 2026 Oct 8;18(10):432. doi: 10.3390/toxins18100432 (PMC13709537; doi:10.3390/toxins18100432)
Supplement: Supplementary file 1 [file toxins-18-00432-s001.zip › toxins-4556637-supplementary.pdf]

# Supplementary Materials: Cardiolipin-Specific Targeting of Melittin via Arg22 in Lipid Membranes: A Natural Toxin-Based Strategy for Treating Mitochondrial Dysfunction in Aging and Disease

**Safy M. Sh. M. Abaza, Jiaqi Zhou, Ziqin Wang, Zanyu Pan, Haoran Ma, Yingdi Sun, Ruben K. Dagda and Edward S. Gasanoff**

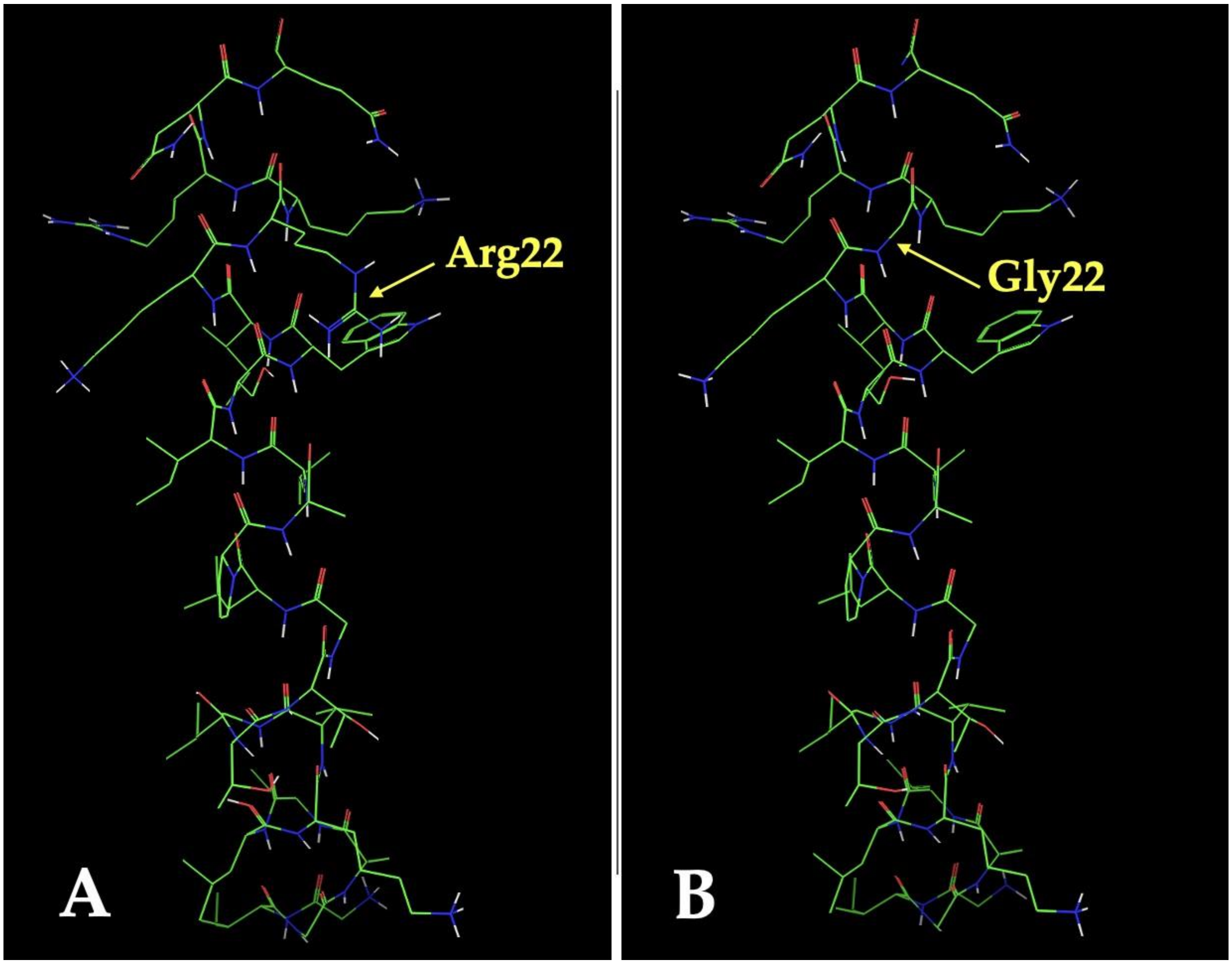


**Figure S1.** Three-dimensional schematic representation of the amino acid sequences of native melittin (A) and modified melittin with Arg22 substituted by Gly22 (B), shown in line representation. Carbon atoms are shown in green, oxygen in red, hydrogen in white, and nitrogen in blue.

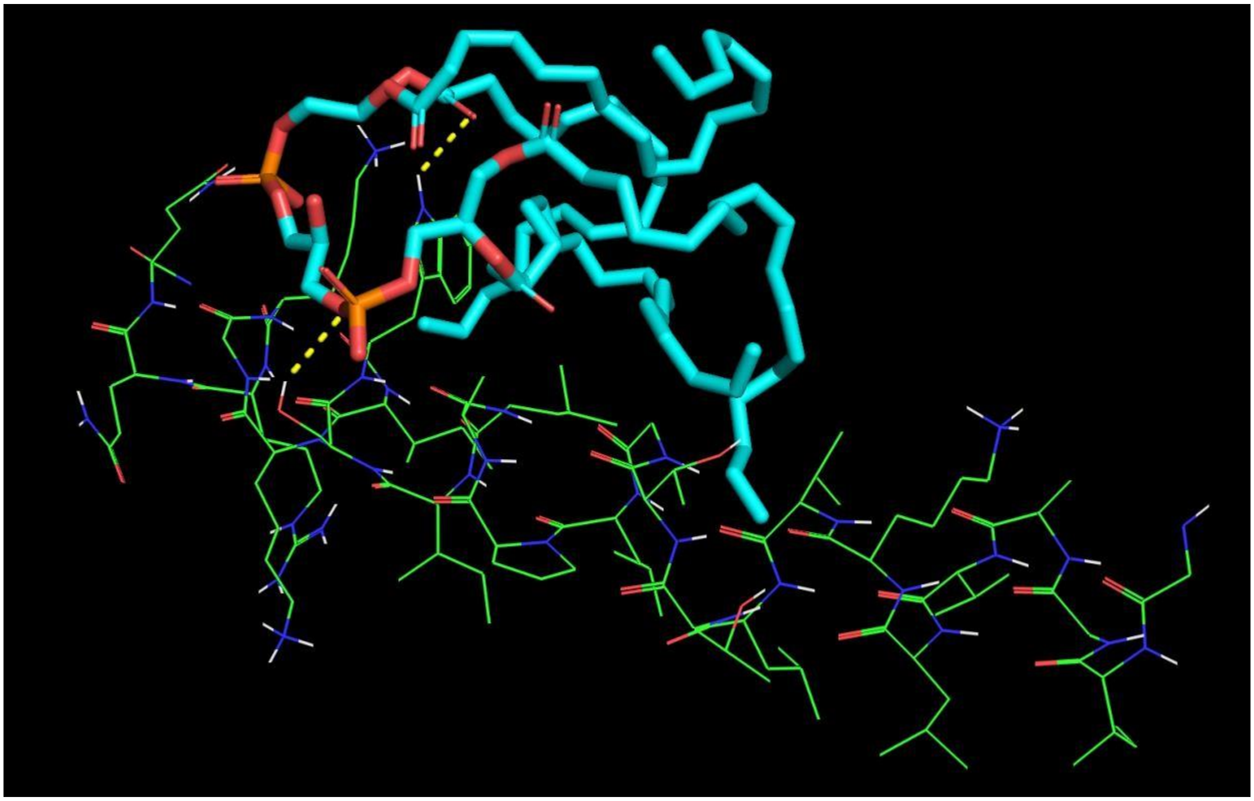

**Figure S2.** Binding site #1 for CL on the molecular surface of modified melittin, as predicted by AutoDock Vina simulation. Modified melittin is shown in line representation, with carbon in green, oxygen in red, hydrogen in white, and nitrogen in blue. CL is rendered in stick representation, with carbon in emerald, phosphorus in orange, and oxygen in red; hydrogen atoms are omitted for visual clarity. Intermolecular bonds are depicted as yellow dashed lines.

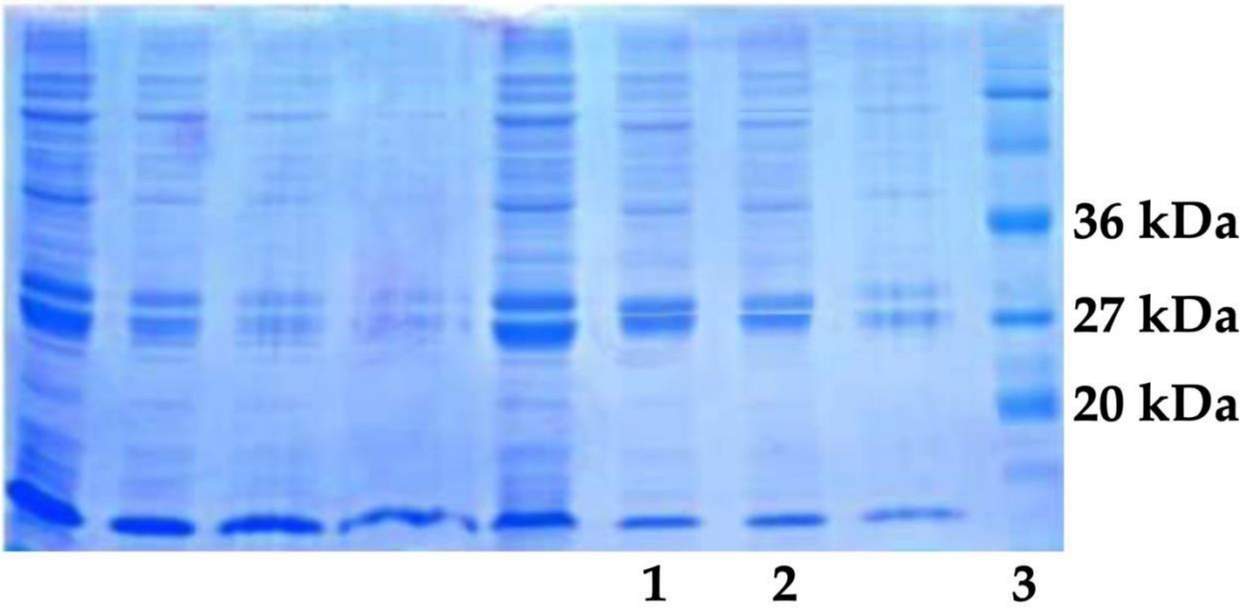


**Figure S3.** SDS-polyacrylamide (14%) gel electrophoresis of eluates from a glutathione Sepharose affinity column eluted with buffer B. Lane 1: eluate containing GST-native melittin; Lane 2: eluate containing GST-modified melittin; Lane 3: molecular weight markers (Takara, Dalian, China). Additional lanes show unrelated supernatant samples from lysed cells filtered through a 0.22 μm microporous membrane; these samples are not individually labeled.

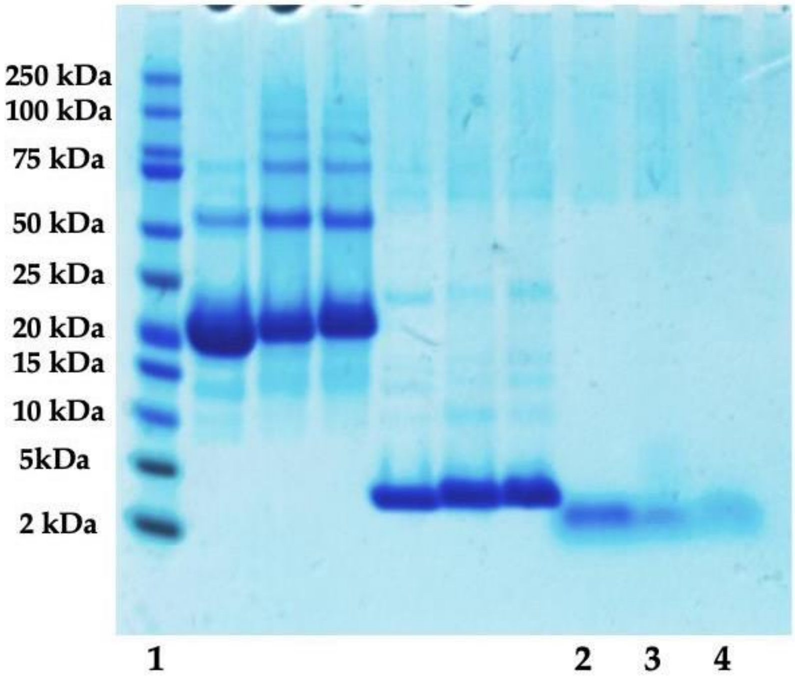


**Figure S4.** SDS-polyacrylamide (18%) gel electrophoresis of r-modified melittin (lane 2), r-native melittin (lane 3) and commercial melittin (98%purity) (lane 4). Lane 1: molecular weight markers from Presicion Plus Protein™ Dual Xtra Standards (Bio-Rad, Carlsbad, CA, USA). Additional lanes, which are not individually labeled, show unrelated samples.

**Table S1.** Adsorption of $H^+$ onto the surface of PC liposomes containing 20 mol% CL, at a total lipid concentration of $6\times10^{-8}$ M and concentration of CL $1.2\times10^{-8}$ M. All pH measurements were taken at 25 °C. The concentration of $H^+$ adsorbed by the liposomes was determined by subtracting the [$H^+$] in the liposome-containing saline solution from that in pure saline solution. The molar ratio of $H^+$ adsorbed by liposomes per $1.2\times10^{-8}$ M of CL is provided in the first sub-column of the last column (in yellow). The first row in the first sub-column of the last column (no color) corresponds to pure liposomes (no melittin), while the subsequent rows represent liposomes with varying molar ratios of CL to bee venom melittin (MLT).

| **pH in saline** | **[$H^+$] in saline, (M)** | **pH in saline with liposomes** | **[$H^+$] in saline with liposomes, (M)** | **[$H^+$] adsorbed by liposomes in saline, (M)** | **Molar ratio of adsorbed protons ($H^+$) per CL*, molar ratio of CL to MLT‡, and MLT‡ concentrations** | | |
|---|---|---|---|---|---|---|---|
| 6.880±0.001 | $1.3180\times10^{-7}$ | 6.896±0.003 | $1.2700\times10^{-7}$ | $0.0480\times10^{-7}$ | 0.4000 | **CL/MLT‡** | **MLT‡ (M)** |
| 6.880±0.001 | $1.3180\times10^{-7}$ | 6.896±0.002 | $1.2700\times10^{-7}$ | $0.0480\times10^{-7}$ | 0.4000 | 200 | $6.00\times10^{-11}$ |
| 6.880±0.001 | $1.3180\times10^{-7}$ | 6.896±0.003 | $1.2700\times10^{-7}$ | $0.0480\times10^{-7}$ | 0.4000 | 170 | $7.05\times10^{-11}$ |
| 6.880±0.001 | $1.3180\times10^{-7}$ | 6.896±0.002 | $1.2700\times10^{-7}$ | $0.0480\times10^{-7}$ | 0.4000 | 140 | $8.57\times10^{-11}$ |
| 6.880±0.001 | $1.3180\times10^{-7}$ | 6.896±0.002 | $1.2700\times10^{-7}$ | $0.0480\times10^{-7}$ | 0.4000 | 110 | $1.09\times10^{-10}$ |
| 6.880±0.001 | $1.3180\times10^{-7}$ | 6.927±0.005 | $1.1836\times10^{-7}$ | $0.1344\times10^{-7}$ | 1.1200 | 80 | $1.50\times10^{-10}$ |
| 6.880±0.001 | $1.3180\times10^{-7}$ | 7.121±0.071 | $1.0167\times10^{-7}$ | $0.3013\times10^{-7}$ | 2.5110 | 50 | $2.40\times10^{-10}$ |
| 6.880±0.001 | $1.3180\times10^{-7}$ | 8.246±0.172 | $0.5680\times10^{-7}$ | $1.2612\times10^{-7}$ | 10.510 | 20 | $6.00\times10^{-10}$ |
| 6.880±0.001 | $1.3180\times10^{-7}$ | 8.439±0.182 | $0.0364\times10^{-7}$ | $1.2816\times10^{-7}$ | 10.680 | 10 | $1.20\times10^{-9}$ |
| 6.880±0.001 | $1.3180\times10^{-7}$ | 8.483±0.212 | $0.0329\times10^{-7}$ | $1.2851\times10^{-7}$ | 10.709 | 5 | $2.40\times10^{-9}$ |

*Molar ratio of adsorbed protons to CL was determined by dividing [$H^+$] adsorbed by liposomes by CL concentration ($1.2\times10^{-8}$ M). ‡Bee venom melittin. Each pH value represents the mean of triplicate measurements ± SD. ANOVA with Tukey's test was used to compare means between groups treated with different melittins and Student's *t*-test was used to compare means for the group treated with the same type of melittin. A $p$-value < 0.05 was considered statistically significant.
